# Primary cilia promote the differentiation of human neurons through the WNT signaling pathway

**DOI:** 10.1186/s12915-024-01845-w

**Published:** 2024-02-27

**Authors:** Andrea Coschiera, Masahito Yoshihara, Gilbert Lauter, Sini Ezer, Mariangela Pucci, Haonan Li, Alan Kavšek, Christian G. Riedel, Juha Kere, Peter Swoboda

**Affiliations:** 1 Department of Biosciences and Nutrition, Karolinska Institute, Huddinge, Sweden; 2 University of Helsinki, Stem Cells and Metabolism Research Program, and Folkhälsan Research Center, Helsinki, Finland; 3 Department of Artificial Intelligence Medicine, Graduate School of Medicine, Chiba, Japan; 4 Present Address: Chiba University, Chiba, Japan; 5 Department of Immunology, Genetics and Pathology, Rudbeck Laboratory, Uppsala, Sweden; 6 Present Address: Uppsala University, Uppsala, Sweden; 7 Department of Bioscience and Technology for Food, Agriculture and Environment, Teramo, Italy; 8 Present Address: University of Teramo, Teramo, Italy

**Keywords:** Primary cilia, WNT signaling, Neuron differentiation, Axon branching, Transcriptomics time-course

## Abstract

**Background:**

Primary cilia emanate from most human cell types, including neurons. Cilia are important for communicating with the cell’s immediate environment: signal reception and transduction to/from the ciliated cell. Deregulation of ciliary signaling can lead to ciliopathies and certain neurodevelopmental disorders. In the developing brain cilia play well-documented roles for the expansion of the neural progenitor cell pool, while information about the roles of cilia during post-mitotic neuron differentiation and maturation is scarce.

**Results:**

We employed ciliated Lund Human Mesencephalic (LUHMES) cells in time course experiments to assess the impact of ciliary signaling on neuron differentiation. By comparing ciliated and non-ciliated neuronal precursor cells and neurons in wild type and in RFX2 -/- mutant neurons with altered cilia, we discovered an early-differentiation “ciliary time window” during which transient cilia promote axon outgrowth, branching and arborization. Experiments in neurons with *IFT88* and *IFT172* ciliary gene knockdowns, leading to shorter cilia, confirm these results. Cilia promote neuron differentiation by tipping WNT signaling toward the non-canonical pathway, in turn activating WNT pathway output genes implicated in cyto-architectural changes.

**Conclusions:**

We provide a mechanistic entry point into when and how ciliary signaling coordinates, promotes and translates into anatomical changes. We hypothesize that ciliary alterations causing neuron differentiation defects may result in “mild” impairments of brain development, possibly underpinning certain aspects of neurodevelopmental disorders.

**Supplementary Information:**

The online version contains supplementary material available at 10.1186/s12915-024-01845-w.

## Background

The primary cilium is an antenna-like structure projecting off polarized cell surfaces. It contains and elongates a microtubule-based axoneme from a modified mother centriole, the basal body, which is paired with a daughter centriole. The pair of centrioles is also part of the centrosome, a major cellular microtubule-organizing center (MTOC) important for mitosis [1], restricting ciliogenesis to the post-mitotic G1 (or G0) phase of the cell cycle and cilia disassembly to prior to the next mitosis [2].

Ciliogenesis is regulated by FOXJ1 and by members of the RFX transcription factor (TF) family [3–5], and mutations in these ciliogenic genes cause ciliary phenotypes [6, 7]. In human there are eight RFX genes: *RFX1-4* and *RFX7* are widely expressed, including in brain tissues and the spinal cord [8], *RFX2* is broadly required for ciliogenesis during vertebrate development [9, 10].

Primary cilia emanate from most human cell types, including neurons. Cilia are formed by the intraflagellar transport (IFT) machinery consisting of protein complexes that convey cargo bidirectionally along the microtubule-based ciliary shaft [11]. Cilia are important for communicating with the cell´s immediate environment: signal reception and transduction to/from the ciliated cell [12]. Cilia harbor signal reception and transduction proteins: they coordinate and transduce a variety of essential signaling pathways [13]. Among these, WNT signaling plays a key role during embryogenesis and adult tissue homeostasis [14]. Canonical and non-canonical WNT signaling is activated by distinct endogenous and exogenous ligands [15]. The main mediator of canonical WNT signaling is β-catenin, which accumulates in the cytoplasm and then translocates to the nucleus. There, β-catenin activates T-cell factor/lymphoid enhancer factor (TCF/LEF) TFs, in turn affecting target / WNT signaling output gene expression [16]. Non-canonical, β-catenin independent WNT signaling activates the cytoplasmic β-catenin destruction complex, and thereby a different TF output complex called Activator protein 1 (AP-1) [17, 18]. Through its interaction with the β-catenin destruction complex the ciliary protein Inversin modulates the balance of WNT pathways by tipping it toward non-canonical WNT signaling [19, 20]. A switch from canonical to non-canonical WNT signaling has been reported to mediate neural stem cell differentiation [21] and cilia may be the main cellular organelle initiating that switch.

Defective cilia can lead to conditions known as ciliopathies, which display pleiotropic clinical, including brain, phenotypes [22, 23]. Also, certain neurodevelopmental conditions and disorders such as dyslexia [24, 25], autism and schizophrenia [11, 26], display strong connections to ciliary aberrations, including through disease-associated candidate genes with demonstrated ciliary functions.

During embryogenesis, cilia extend apically from the neuroepithelium into the lumen of the neural tube to detect morphogens gradients [27]. Cilia direct the transition of neuroepithelial stem cells into neural progenitor cells (neurogenesis) that will give rise to all neuronal and glial cell types of the central nervous system [28]. Cilia as sensory and signaling devices have well-documented roles in the initial expansion of the neural progenitor cell pool [22, 28], and in neuron migration [29, 30]. Also, recent reports demonstrated that cilia impact axon navigation [31], growth cone and axon tract development (PI3K/AKT signal transduction, [32], the shaping of dendritic structures (AC3 signal transduction [33], and interneuronal connectivity (GPCR signaling [34].

In parts concurrent with neuron migration, neuron differentiation follows a pattern of successive stages, encompassing precursor cell polarization, axonogenesis, axon outgrowth, elongation and branching, the formation of dendrites, spines and synapses, including pruning steps, and finally the formation of neuronal circuits [35–37]. The impact of cilia on (early) neuron differentiation is still under debate, while centrosome and Golgi apparatus have already been shown to be crucial for microtubule nucleation during polarization after mitosis and for axon specification and axonogenesis [36, 37].

Here we investigate cilia in a human neuronal cell line, LUHMES [38], where cells proliferate as neuronal precursors or can be induced to differentiate and mature into neurons. Upon induction to differentiate, ciliation occurs asynchronously, is transient during the early differentiation stages and not every neuron ciliates. We determined in individual neurons how and when cilia impact neuron differentiation, by comparing ciliated and non-ciliated neurons in wild type (WT) and when cilia are structurally and functionally altered (using a ciliogenic RFX2 -/- TF mutant and knockdowns of the essential ciliary genes *IFT88* and *IFT172*). We discovered a “ciliary time window” during which cilia promote axon outgrowth, branching and arborization. Cilia promote these neuron differentiation steps by tipping WNT signaling toward the non-canonical pathway, in turn activating WNT pathway output genes implicated in cyto-architectural changes. Thereby we provide a mechanistic entry point into how and when during neuron differentiation ciliary signaling coordinates, promotes and translates into anatomical changes. The emerging, appropriate neuron anatomy is a prerequisite for establishing functional connections (circuit formation). Our findings suggest a critical spatiotemporal role of cilia in neuron and brain development. We hypothesize that ciliary alterations causing neuron differentiation defects may result in “mild” impairments of brain development, possibly underpinning the onset of certain aspects of neurodevelopmental disorders.

## Results

### Cilia promote neuron differentiation: axon outgrowth

In the LUHMES cell model [26], we used unsynchronized neuronal precursor cell proliferation conditions and timed (day 0/day 1; d0/d1), change of culture medium-induced release into neuron differentiation. We noticed that neurons with detectable cilia more efficiently accomplished axon outgrowth as compared to non-ciliated neurons.

**Fig. 1:**
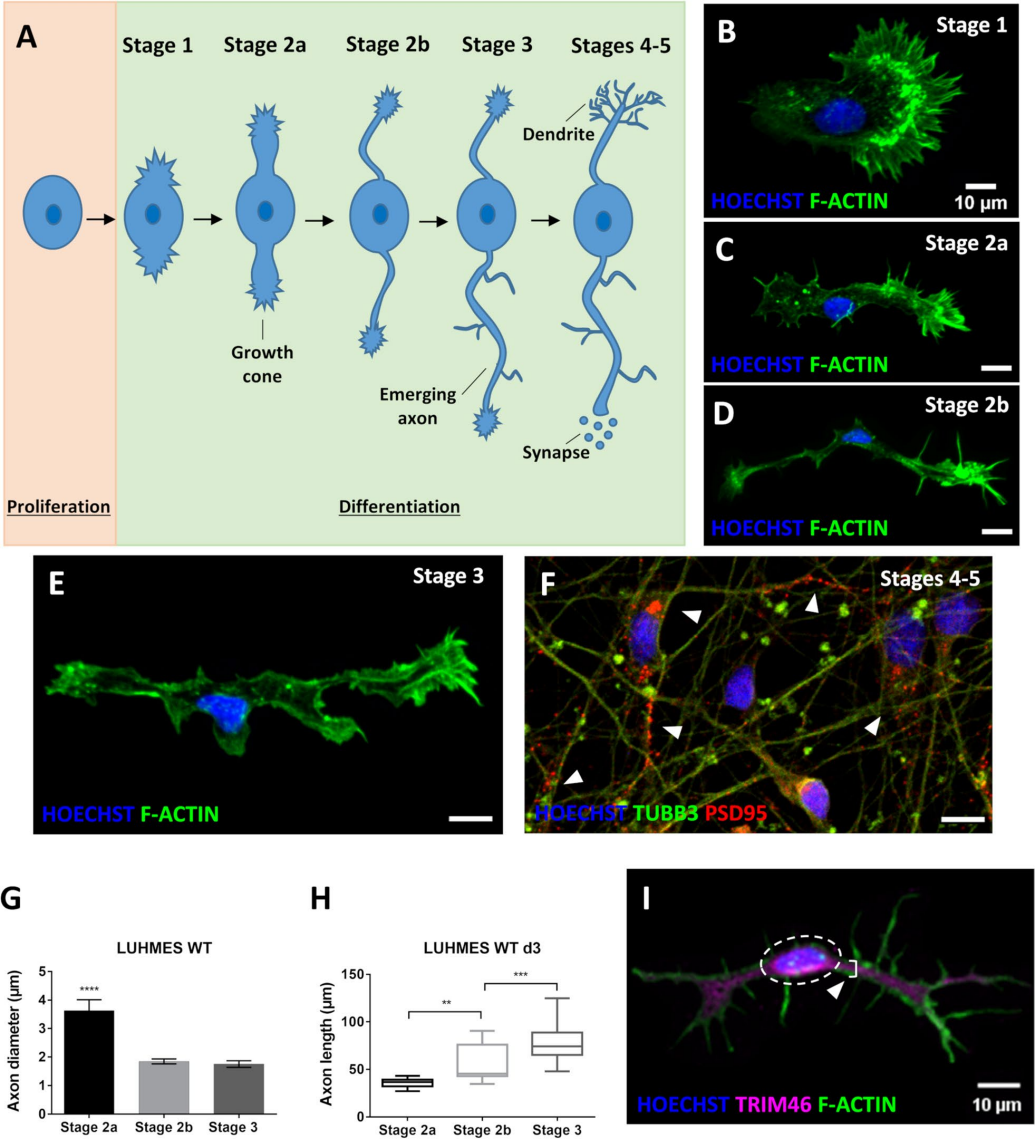
Stages of neuron differentiation: cilia promote axon outgrowth. **A** Schematic illustration of neuron differentiation stages [36]: stage 1 – growth cone protrusions during neuron polarization; stage 2a – symmetric bipolar stage with microtubule invasion into growth cones, first neurite extension; stage 2b – symmetric bipolar stage with microtubule bundles consolidation, further neurite extension; stage 3 – the emerging axon outgrows the opposite neurite breaking the bipolar symmetry; stages 4 and 5 – dendrites and dendritic spines formation, further maturation with formation of synapses. **B**-**F** Staging of human LUHMES neuron differentiation: immunocytochemistry detects nuclei (Hoechst staining), the cytoskeleton (Phalloidin stains F-actin), microtubule bundles (TUBB3) and emerging synapses (PSD95).** G** Neurons at differentiation stage 2a display a significantly larger diameter of the emerging axon than neurons at stages 2b and 3 (*n* = 20–26). **H** Axon elongation between differentiation stages 2a, 2b and 3 (*n* = 21–37). **I** Phalloidin staining is used to identify the cell body (dashed line) and the axon marker TRIM46 to recognize the initial segment of the axon (arrowhead and bracket), the diameter of which was used for measurements. Mean values are shown ± s.e.m. (**G**) and displayed as Box and Whisker plots (min to max) (**H**). The results are from two independent experiments including two technical replicates each. We conducted regular one-way ANOVA analyses with multiple comparisons (Bonferroni’s test) between groups. **p < 0.005; ***p < 0.0005; ****p < 0.0001

To quantify these differentiation differences, we performed stringent time course experiments, where we assessed established neuron differentiation and outgrowth milestones: stage 1 (growth cone protrusions), stages 2a and 2b (bipolar stage, engorgement and consolidation), stage 3 (axon outgrowth, break of symmetry), and stages 4 and 5 (maturation, formation of dendrites, spines and synapses) [36, 37] (Fig. 1A). LUHMES neurons differentiate through these stages within about one week [26] (Fig. 1B-F). We performed immunocytochemistry using markers for cilia (ARL13B), centrosomes/basal bodies (PCNT) and for axons (TAU, TRIM46) [39–41] to quantify the presence of cilia and emerging axons (stage 3) throughout the entire LUHMES neuron differentiation protocol (d0-d6).

**Fig. 2:**
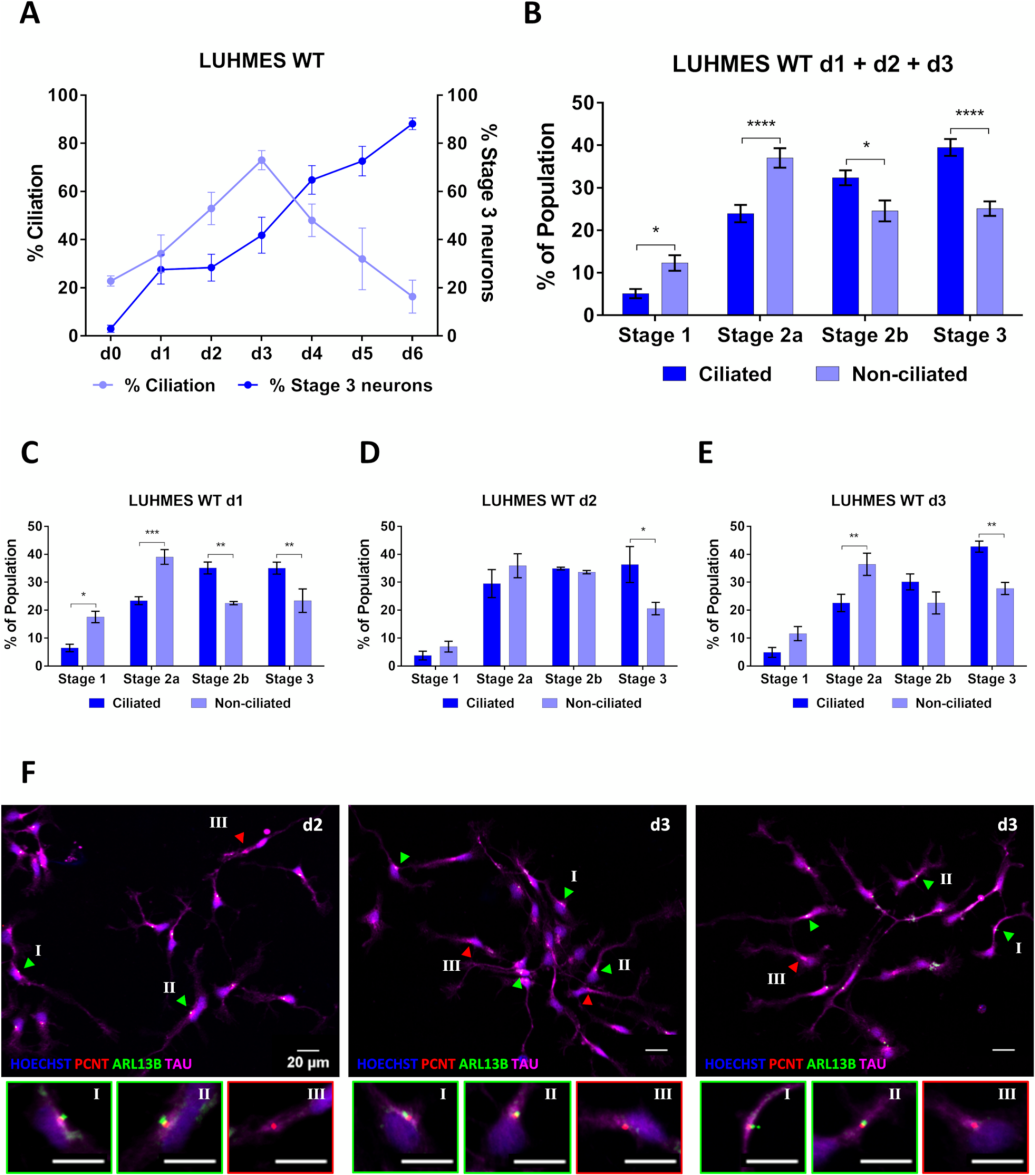
Differentiation advantage of ciliated neurons as compared to non-ciliated neurons in a LUHMES WT background. **A** The percentage of ciliation in differentiating human LUHMES neurons increases once neurons exit the cell cycle (d1), peaks in differentiating neurons (d3) and then decreases during the later stages of neuron differentiation and maturation (d4-d6). The percentage of neurons reaching stage 3 of differentiation steadily increases throughout the entire differentiation and maturation process (n = 134–394). **B**-**E** Populations of ciliated LUHMES neurons (*n* = 39–128) are more efficient than non-ciliated LUHMES neurons (*n* = 15–116) in breaking the bipolar symmetry of stages 2a and 2b, and thus, in promoting axon outgrowth (d1-d3 summarized and individual days). **F** Identification by immunocytochemistry of nuclei (Hoechst staining), centrosomes/basal bodies (PCNT marker), cilia (ARL13B marker) and axons (TAU marker) reveals that the majority of stage 3 differentiating neurons in a given population are ciliated. Arrowheads point to stage 3 neurons either being ciliated (in green) or non-ciliated (in red). Mean values ± s.e.m. are shown. The results are from a minimum of three independent experiments with a total of at least six technical replicates. We conducted regular two-way ANOVA analyses (not repeated measures) with multiple comparisons (Bonferroni’s test) between groups. *p < 0.05; **p < 0.005; ***p < 0.0005; ****p < 0.0001

We released LUHMES precursor cells into neuron differentiation from an unsynchronized cell cycle starting point (d0/d1). Thereby some cells exit the cell cycle, become post-mitotic and ciliate earlier than others during the initial phases of differentiation. Ciliation increased steadily during early differentiation, peaking at d3 when most neurons (about 70%) are ciliated. Detectable ciliation then decreased during the later phases of differentiation (d4-d6) (Fig. 2A). For each differentiation time point (d0-d6), we also determined the number of neurons in the population that were able to break the bipolar symmetry and initiate the outgrowth of the future axon (stages 2 and 3). The percentage of neurons with a defined, emerging axon steadily increased over time, consistent with the ongoing maturation of neurons during the differentiation process (Fig. 1G-I, 2A). At a population level, ciliation “precedes” axon outgrowth (Fig. 2A).

In these populations of differentiating LUHMES neurons we then compared ciliated and non-ciliated neurons regarding how efficiently they reach differentiation stage 3 during the initial phases of the differentiation process (d1-d3). We found that the majority of neurons that reach differentiation stages 2b and 3 were neurons with a cilium, while for the earlier differentiation stages 1 and 2a the non-ciliated neuron subpopulation was in the majority, at all three time points examined (d1-d3) (Fig. 2B-F, Additional file 1: Table S1).

Taken together our results indicate a strong correlation between the presence of cilia and an accelerated progression through successive neuron differentiation stages during the initial phases, constituting a “transient ciliary time window”, exemplified by a crucial feature, break of symmetry and axon outgrowth.

### Cilia structure and function are altered in human LUHMES RFX2 -/- (knockout) cells and neurons

To experimentally explore these differences in differentiation between ciliated and non-ciliated neurons, we altered cilia by mutation. We knocked out the gene for the ciliogenic transcription factor (TF) RFX2. The RFX family of TFs is essential for ciliogenesis [6]. In vertebrates *RFX2* gene function is required for ciliogenesis during development [10], in humans *RFX2* is abundantly expressed in the brain [8], and in human LUHMES neurons *RFX2* is highly expressed during early differentiation (d0-d2/d3) [26].

**Fig. 3:**
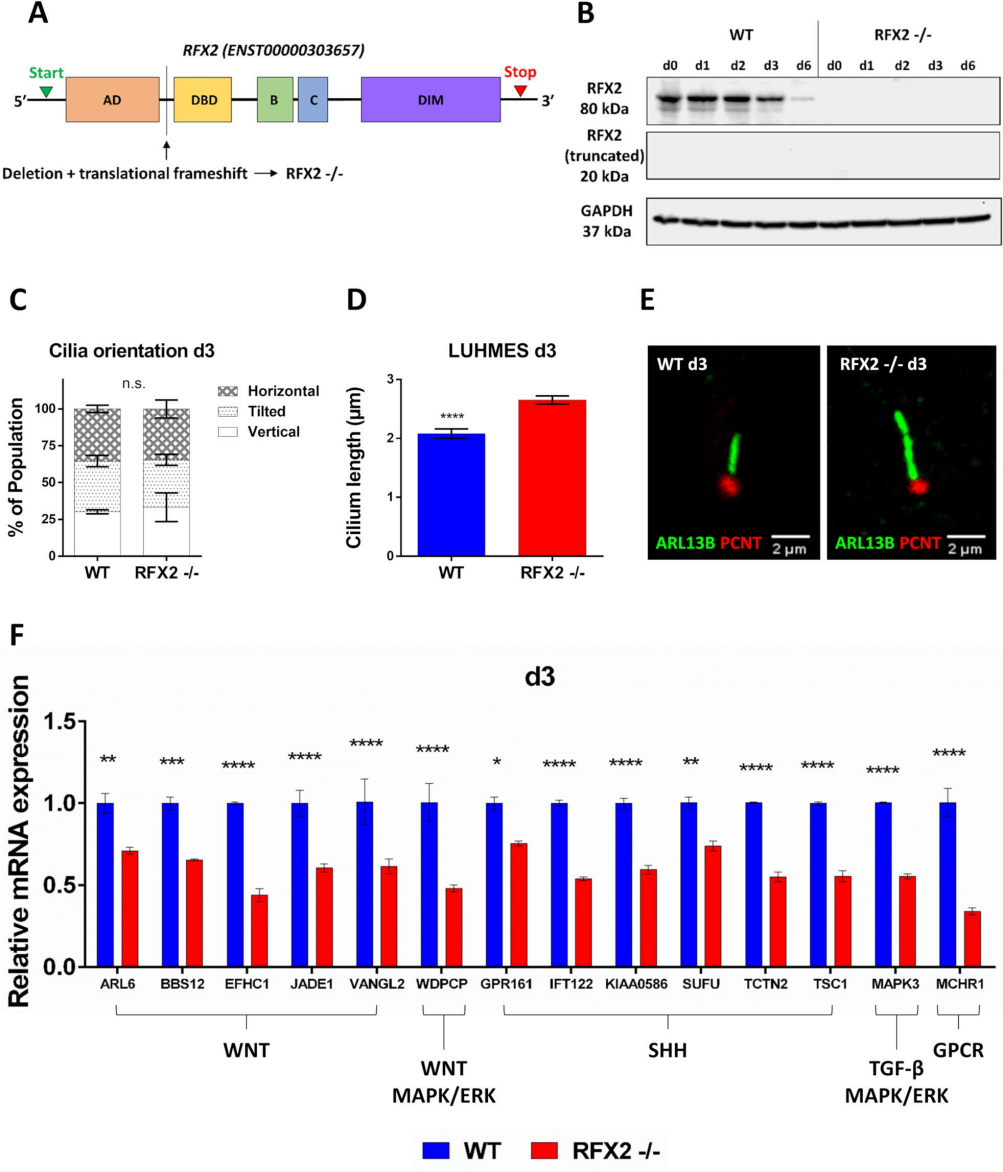
Cilia structure and function are altered in human LUHMES RFX2 -/- mutants. See also Additional file 2: Fig. S1. **A** Schematic illustration of the RFX2 transcription factor (TF) gene structure with functional protein domains indicated: AD – activation domain, DBD – DNA binding domain, B and C – domains B and C of unknown function, DIM – dimerization domain. CRISPR/Cas9-engineered, small deletions lead to reading frame shifts upstream of the RFX2 DBD, essential for TF functionality, thereby creating an RFX2 -/- knockout mutant. **B** Western blot analyses of RFX2 protein expression in LUHMES wild-type (WT) and RFX2 -/- mutants: In the RFX2 -/- mutant RFX2 protein expression is completely absent during early neuron differentiation time points (d1-d3) and at the end of differentiation and maturation (d6). Also, no protein expression of a hypothetical, predicted truncated RFX2 protein product was detectable because of CRISPR/Cas9 mutagenesis. The housekeeping protein GAPDH was used as a loading control. **C** WT (*n* = 171) and RFX2 -/- (*n* = 215) neuronal populations display a similar and uniform distribution of cilia orientation in 3D space. **D** Cilia of RFX2 -/- mutants are longer than WT cilia in differentiating neurons (d3). Immunofluorescence-based length measurements: ARL13B marks the ciliary shaft, PCNT marks the basal body. Statistics: WT (*n* = 76), RFX2 -/- (*n* = 111). **E** Representative immunocytochemistry images of WT vs elongated RFX2 -/- cilia. **F** Ciliary signaling pathway genes relevant for neuron differentiation are deregulated between differentiating WT and RFX2 -/- neurons. Mean values ± s.e.m. are shown from two independent experiments with a minimum of three technical replicates each. We conducted regular one-way ANOVA (**D**) and two-way ANOVA analyses (**C**, **F**) (not repeated measures) with multiple comparisons (Bonferroni’s test) between groups. *p < 0.05; **p < 0.005; ***p < 0.0005; ****p < 0.0001

We used CRISPR/Cas9 with guide RNAs (gRNAs) specific for sequences upstream of the exons encoding the RFX2 DNA binding domain (DBD), essential for protein (TF) function. Thereby we generated LUHMES RFX2 -/- deletion alleles that lead to translational frame shifts and premature stop codons, destroying the DBD and all subsequent protein domains, thus constituting a gene knockout mutation (Fig. 3A, Additional file 2: Fig. S1A-D). We demonstrated by qRT-PCR that *RFX2* mRNA expression is strongly downregulated (Additional file 2: Fig. S1B), and by Western blot that RFX2 protein is absent (Fig. 3B, Additional file 3: Fig. S1A), throughout the entire neuron differentiation time course.

In control experiments we then evaluated whether the RFX2 -/- genotype caused any overt growth or gross anatomical abnormalities in differentiating neurons. We found this not to be the case. Both WT and RFX2 -/- neurons displayed near-identical growth rates, whether grown in regular growth medium for the proliferation of the precursor cell stage or when grown in growth and differentiation medium for the induction of neuron differentiation (Additional file 2: Fig. S1E). Neither did we find any relevant differences between WT and RFX2 -/- neurons in overall neuron anatomy during the early phases of differentiation (d1-d3). Using whole-cell immunocytochemistry (neurites were marked with either anti-TAU or anti-TRIM46) we detected that both cultures were dominated to near-identical extents by bipolar, followed by multipolar and then unipolar neurons (Additional file 2: Fig. S1F-H).

To determine the impact of the RFX2 -/- mutations on ciliary structure we used confocal microscopy. First, we confirmed that there was no difference in cilia orientation and distribution in 3D space (horizontal, tilted, vertical) between WT and RFX2 -/- neurons (Fig. 3C) [42]. Then we determined ciliary length, which we found to be significantly longer in RFX2 -/- than in WT (Fig. 3D-E). Aside from structural alterations, mutation of RFX2 led to a significant downregulation, as assessed by qRT-PCR, of ciliary genes [43], including components of the following signaling pathway genes relevant for neuron differentiation: WNT (*ARL6, BBS12, EFHC1, JADE1, VANGL2, WDPCP*), SHH (*GPR161, IFT122, KIAA0586, SUFU, TCTN2, TSC1*), TGF-β (*MAPK3*), GPCR (*MCHR1*) and MAPK/ERK (*WDPCP, MAPK3*) (Fig. 3F). Taken together, our observations, expectedly, point toward a ciliary role of RFX2, while the cell cycle and cell proliferation, and gross neuron anatomy appear to be unaffected by mutation of RFX2.

### Absent or altered cilia affect different aspects of human LUHMES neuron differentiation

Our experimental setup allowed for direct comparisons between differentiating neurons: ciliated *versus* altered cilia *versus* non-ciliated. Using immunocytochemistry, we focused on two different axon-associated phenotypes during the entire time course of neuron differentiation and maturation (d0-d6): outgrowth and branching.

**Fig. 4:**
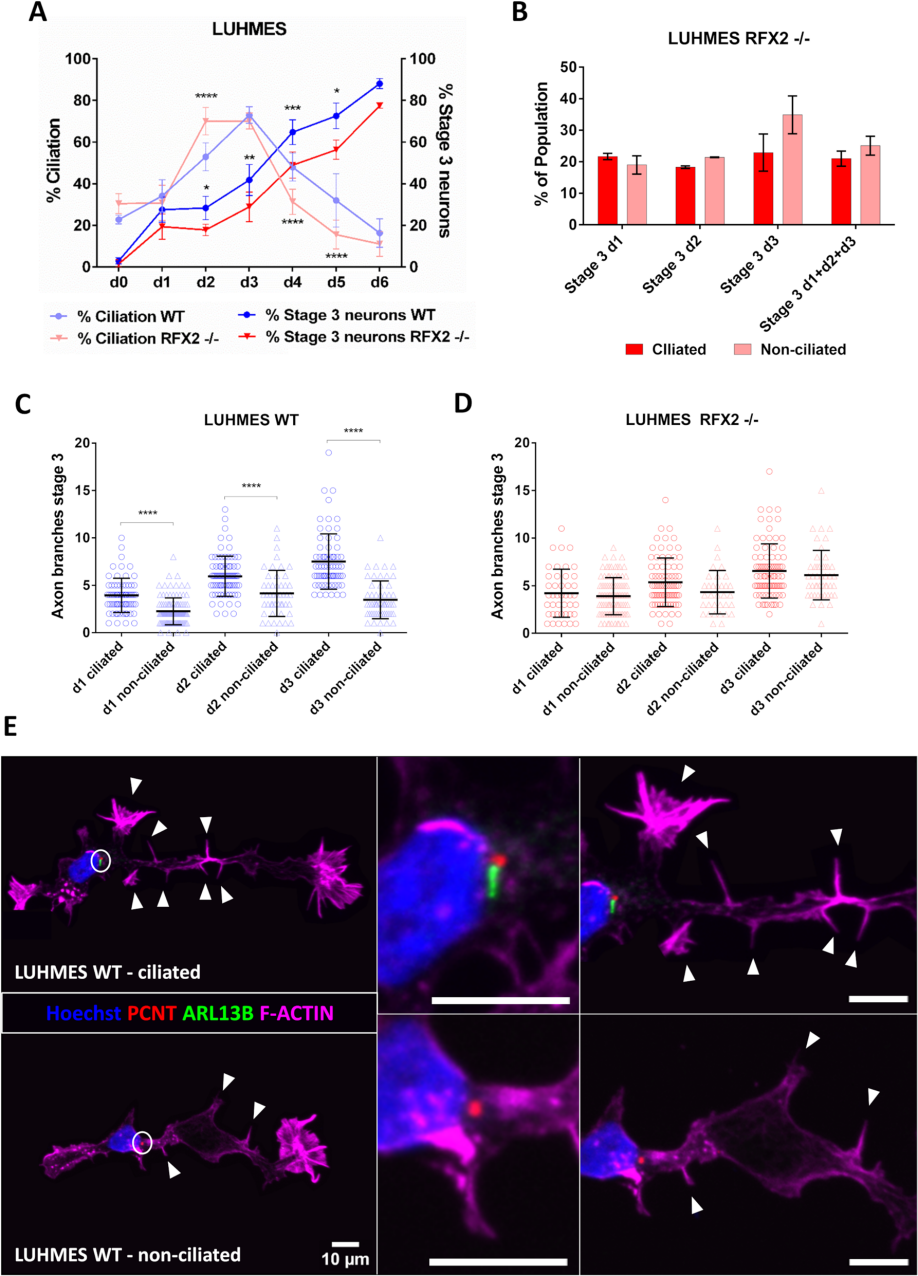
Altered cilia affect different aspects of human LUHMES neuron differentiation: axon outgrowth and branching pattern. **A** The percentage of ciliation in differentiating human LUHMES neurons increases and declines slightly earlier in RFX2 -/- mutants as compared to WT neurons. The percentage of neurons reaching stage 3 of differentiation is delayed in RFX2 -/- mutants as compared to WT neurons throughout the entire differentiation and maturation process (WT *n* = 134–394; RFX2 -/- *n* = 162–492). **B** Altered cilia of LUHMES RFX2 -/- neurons do not render axon outgrowth more efficient (*n* = 29–90), as compared to non-ciliated LUHMES RFX2 -/- neurons (*n* = 24–122) (d1-d3 summarized and individual days). **C**-**D** Axons of ciliated neurons (*n* = 69–80) are more branched and arborized as compared to non-ciliated neurons *n* = 48–107 in WT, whereas in RFX2 -/- mutants there is no difference between ciliated (*n* = 43–84) and non-ciliated neurons *n* = 34–103). **E** Detection by immunocytochemistry demonstrates the effect the cilium imparts on the complexity of axon branching in WT neurons: top – ciliated neuron, bottom – non-ciliated neuron (circles indicate centrosome with or without a cilium; arrowheads indicate axon branch points); Hoechst stain – nucleus, PCNT – centrosome/basal body marker, ARL13B – ciliary marker, Phalloidin – cytoskeleton/F-actin marker. Mean values are shown ± s.d. (**A**, **C**-**D**) and ± s.e.m. (**B**). The results are from a minimum of three independent experiments with a minimum of two technical replicates each. We conducted regular two-way ANOVA analyses (not repeated measures) with multiple comparisons (Bonferroni’s test) between groups. *p < 0.05; **p < 0.005; ***p < 0.0005; ****p < 0.0001

Using a marker for cilia (ARL13B) we observed that ciliation was similarly transient for both WT and RFX2 -/- (Fig. 4A). Using markers for axons (TAU, TRIM46) we found consistently that a population of WT neurons more efficiently reached differentiation stage 3 as compared to a population of RFX2 -/- neurons (Fig. 4A). It appears that (functional) WT cilia provide a differentiation “advantage” over altered (RFX2 -/-) or absent cilia to facilitate axon outgrowth (Fig. 2B-F, 4A-B, Additional file 1: Table S1). Using Phalloidin staining to detect cytoskeletal F-actin we then investigated aspects of emerging axon tract development such as axon branching and arborization in both ciliated and non-ciliated differentiation stage 3 neurons. We found in WT ciliated neurons the emerging axons to be significantly more branched than in non-ciliated neurons (Fig. 4C, 4E, Additional file 2: Fig. S2A). These differences in axon branching and arborization were absent in RFX2 -/- when comparing neurons with altered and neurons with absent cilia (Fig. 4D, Additional file 2: Fig. S2A).

We conclude that the presence of functional primary cilia impacts two different aspects of neuron differentiation: it promotes axon outgrowth and subsequent axon branching. While mutation of the ciliogenic RFX2 TF gene, altering cilia structure and function, delays and deregulates these aspects of the neuron differentiation process.

### Ciliary alterations by gene knockdown confirm the ciliary regulation of neuron differentiation

To independently confirm the ciliary regulation of axon outgrowth and branching, we knocked down the expression of two different components of the intraflagellar transport (IFT) machinery (IFT88, IFT172), which is essential for cilia formation and function [44].

**Fig. 5:**
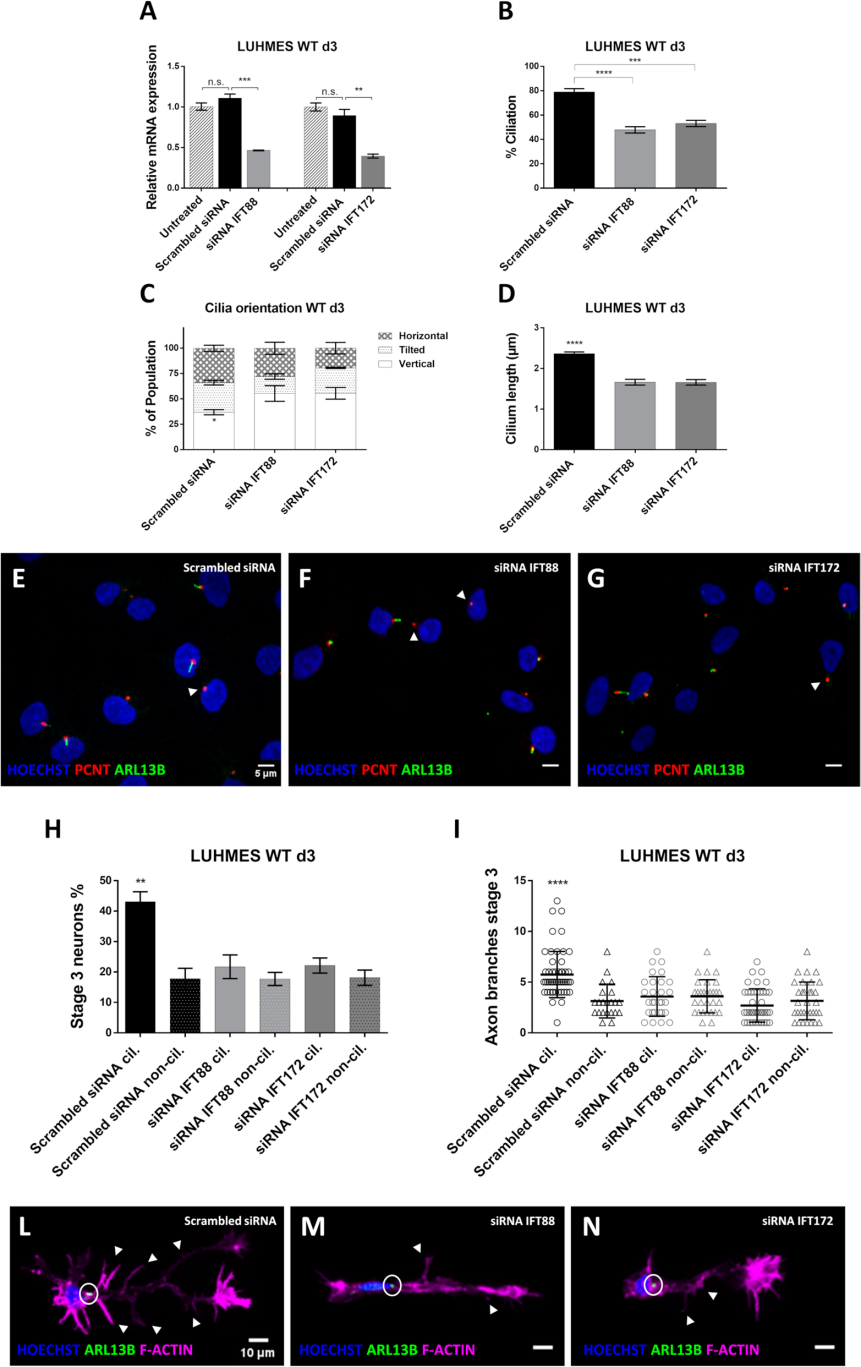
siRNA knockdown of essential ciliary genes *IFT88* and *IFT172* leads to axon outgrowth and branching defects. **A** Gene-specific siRNA significantly downregulates the expression of *IFT88* and *IFT172* as compared to control samples treated with non-specific scrambled siRNA or when untreated. **B** Knockdown (KD) of *IFT88* and *IFT172* expression results in fewer ciliated LUHMES neurons than in the control (scrambled siRNA n = 271; siRNA IFT88 *n* = 314; siRNA IFT172 *n* = 405). **C** Differently from the WT vs RFX2 -/- backgrounds (cf. Fig. 3C), a significantly higher percentage of cilia in *IFT88* KD (*n* = 66) and *IFT172* KD (*n* = 72) appears to be vertically oriented as compared to the control (*n* = 260), strongly suggesting an increased total amount of severely truncated/shorter cilia in horizontal or tilted orientation indistinguishable from the vertical orientation. **D** Cilia length is quantified by the PyT method and confirms that cilia of *IFT88* KD (*n* = 66) and *IFT172* KD (*n* = 72) neurons are shorter than the control ones (*n* = 260). **E**–**G** Immunocytochemistry detection of nuclei (Hoechst staining), centrosomes/basal bodies (PCNT marker) and cilia (ARL13B marker) displays the distribution of vertical (and truncated) cilia in *IFT88* KD and *IFT172* KD neurons. Arrowheads exemplify centrosomes of non-ciliated neurons. **H** Reduced expression of *IFT88* and *IFT172* leads to axon outgrowth defects of ciliated LUHMES neurons (scrambled siRNA *n* = 217; siRNA IFT88 n = 148; siRNA IFT172 *n* = 218), similar to non-ciliated neurons (scrambled siRNA *n* = 54; siRNA IFT88 n = 166; siRNA IFT172 *n* = 187). **I** Axons of *IFT88* KD (*n* = 29) and *IFT172* KD (*n* = 43) ciliated neurons are significantly less branched than the axons of control neurons (n = 55), similar to the axons of non-ciliated neurons (scrambled siRNA *n* = 22; siRNA IFT88 *n* = 30; siRNA IFT172 n = 33). **L**-**N** Representative images of immunocytochemistry of axon branching defects in *IFT88* KD and *IFT172* KD ciliated neurons (circles localize primary cilia; arrowheads point to axon branches); Hoechst staining – nucleus, ARL13B – ciliary marker, Phalloidin – cytoskeleton/F-actin marker. Mean values are shown ± s.d. (**I**) and ± s.e.m. (**A**-**D**,** H**). The results are from two independent experiments with a minimum of three technical replicates each. We conducted regular one-way ANOVA (**A**-**B, D**) and two-way ANOVA analyses (**C, H**-**I**) (not repeated measures) with multiple comparisons (Bonferroni’s test) between groups. *p < 0.05; **p < 0.005; ***p < 0.0005; ****p < 0.0001

Using siRNA-mediated gene knockdown (KD), we caused a significant downregulation of *IFT88* and *IFT172* gene expression (Fig. 5A), as compared to WT (untreated) or WT transfected with non-complementary scrambled siRNA control sequences. On d3 of LUHMES neuron differentiation (at the peak of ciliation), gene knockdown of *IFT88* and *IFT172* resulted in fewer ciliated neurons (Fig. 5B), altered cilia orientation and distribution in 3D space (Fig. 5C, 5E-G), and in significantly shorter cilia (Fig. 5D) as compared to control neurons. We surmise that some of the truncated *IFT88* and *IFT172* KD cilia are microscopically indistinguishable from those oriented vertically in 3D space (cf. [42]). Like in RFX2 -/- (and in non-ciliated) neurons, altered cilia in *IFT88* and *IFT172* KD neurons led to fewer neurons in the population being able to reach differentiation stage 3 (Fig. 5H), confirming that functional cilia are required for promoting axon outgrowth. In accordance with previous work [32], we found axons of ciliated and non-ciliated *IFT88* and *IFT172* KD neurons to be less branched than those of ciliated control neurons (Fig. 5I-N). Interestingly, this is a different phenotype to what we observed in RFX2 -/- neurons where altered cilia are longer than in WT (Fig. 3D, E) and where axons in RFX2 -/- ciliated neurons were as branched as in WT ciliated neurons (Fig. 4C,D, Additional file 2: Fig. S2A). These results further support the notion that axon outgrowth and branching are modulated independently and that functional cilia are crucial to properly promote both aspects of neuron differentiation.

With this independent approach (siRNA knockdown of the essential ciliary genes *IFT88* and *IFT172*) we confirm that functional cilia are necessary for proper neuronal axon outgrowth and branching, as we demonstrated in RFX2 -/- knockout mutant neurons (Fig. 4).

### RFX2 -/- knockout leads to delayed neuron differentiation also at the transcriptome level

**Fig. 6:**
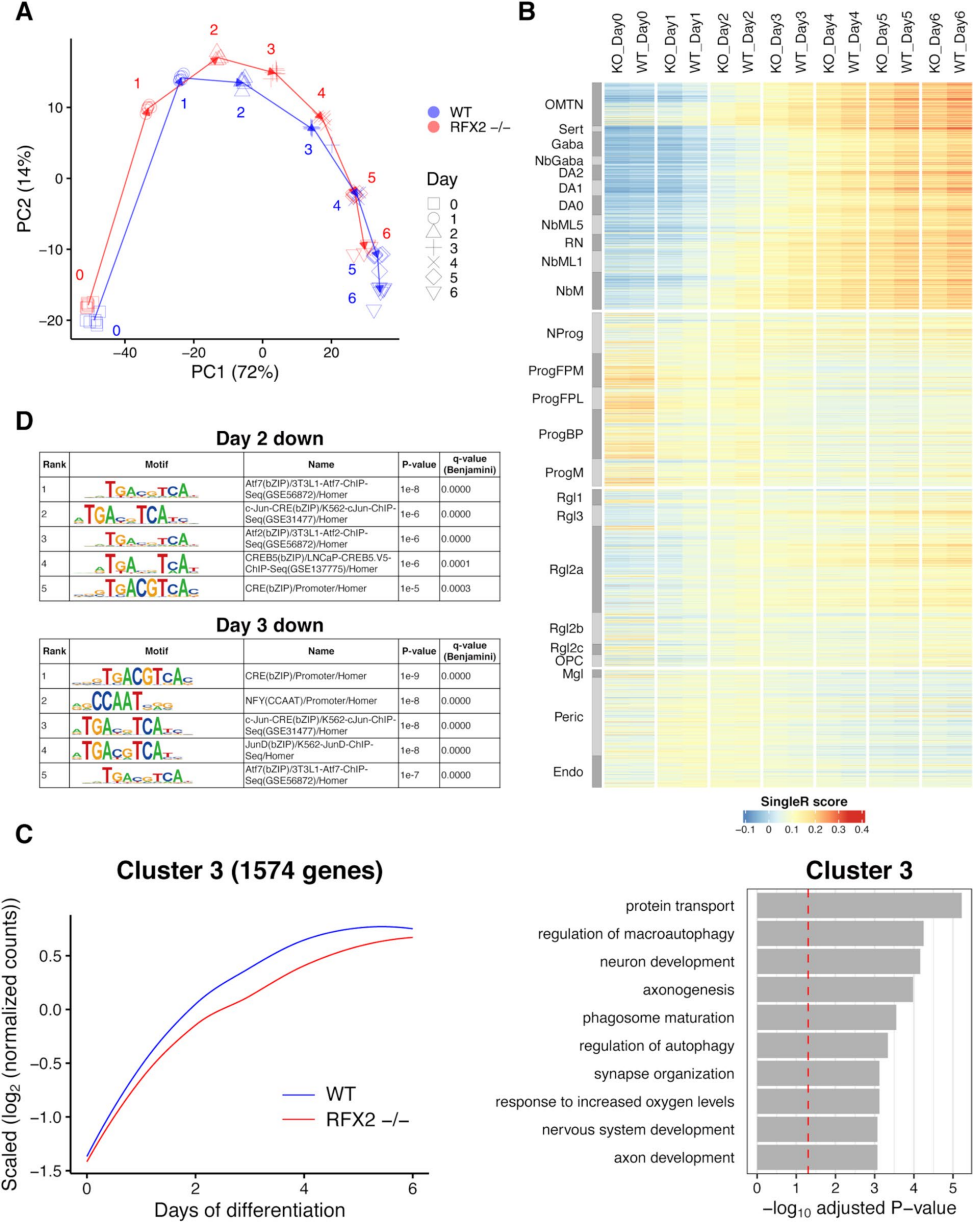
Transcriptomics confirm the delay in differentiation of human RFX2 -/- LUHMES neurons. See also Additional file 2: Fig. S3-S5. **A** Principal component analysis of 78 (41 WT and 37 RFX2 -/-) LUHMES STRT RNA-seq time course samples, representing the process of neuron differentiation from d0 to d6. Numbers represent the days and arrows connect the neighboring days. **B** Similarity heat map of SingleR annotation scores of different types of human fetal midbrain cells and neurons [48] using the 78 LUHMES WT and RFX2 -/- samples as reference. Note the apparent differentiation delay of RFX2 -/-, visible in the comparisons to differentiating neuronal cell types (d1/d2-d6,upper right corner), while in the comparisons to precursor cell types (d0; middle left) no differences between WT and RFX2 -/- are apparent. **C** Left: Scaled expression profile of 1,574 genes downregulated in RFX2 -/- LUHMES STRT RNA-seq samples (Cluster 3) throughout the differentiation time course. 3,476 genes with a significantly variable expression between WT and RFX2 -/- throughout the time course were clustered into three clusters based on their expression patterns during the differentiation process. Lines are local polynomial regression fittings of the scaled expression of the genes in each cluster, depicted in blue (WT) and in red (RFX2 -/-). Right: Gene ontology (GO) enrichment analysis of these 1,574 genes in Cluster 3. The red dashed line represents the adjusted *p*-value = 0.05. Clusters 1–3 are depicted side-by-side in Additional file 2: Fig. S3. **D** Transcription factor binding motif enrichment analysis of significantly downregulated transcript far 5’ ends (TFEs) in RFX2 -/- LUHMES STRT RNA-seq samples at day 2 and day 3: only the top five significantly enriched motifs are listed. The complete transcription factor binding motif enrichment analysis throughout the time course of neuron differentiation and maturation (d0-d6) is shown in Additional file 2: Fig. S5

To monitor gene expression during neuron differentiation, we subjected WT and RFX2 -/- LUHMES cells and neurons to modified single-cell tagged reverse transcription RNA-sequencing (STRT RNA-Seq), which captures the 5’ ends of transcripts (transcript far 5’ ends, TFEs) [45–47]. Cells were collected from d0 to d6 with 4–6 replicates per time point (Additional file 1: Table S2). Principal component analysis (PCA) revealed that RFX2 -/- neurons showed a delayed differentiation pattern as compared to WT. For example, the gene expression profile of d4 differentiating RFX2 -/- neurons displayed high similarity with d3 differentiating WT neurons (Fig. 6A). For confirmation, we compared our STRT RNA-seq results with the single-cell RNA-seq data set from human fetal midbrain in vivo [48]. As shown in a previous study [38], during the time course of differentiation (d0-d6) WT LUHMES neurons progressively acquired high similarity with differentiated human midbrain neurons. RFX2 -/- neurons, on the other hand, showed a pronouncedly weaker similarity with differentiating human midbrain neurons as compared to WT, and again, typically were a day “behind” in differentiation (Fig. 6B).

To identify the temporal gene expression pattern between WT and RFX2 -/-, we clustered 3,476 significantly variable genes into 3 clusters based on the expression pattern over the time course (Fig. 6C, Additional file 2: Fig. S3). Genes in Cluster 3 were upregulated during neuron differentiation but lowly expressed in RFX2 -/- as compared to WT throughout the entire time course. Of note, these 1,574 genes in Cluster 3 were enriched with genes related with neuron development and axonogenesis (Fig. 6C). *DCX* and *MAPT* (neuronal marker genes), *PAX6*, *TH*, and *SST* (neuronal function-related genes) were classified into Cluster 3 (Additional file 2: Fig. S4A, S4B, Additional file 1: Tables S3, S4). The gene ontology (GO) term ‘cilium assembly’ was also significantly enriched in this cluster (adjusted *P*-value = 0.005) (Additional file 1: Table S4). Prominent ciliary genes such as *IFT27*, *TUBB4A*, and *SEPT3* were classified into this cluster (Additional file 2: Fig. S4C, Additional file 1: Tables S3, S4). Further, we found that significantly downregulated genes in RFX2 -/- at each time point were enriched for the term ‘cilium assembly’ and ciliary genes, especially at d3 and d4 of neuron differentiation (Additional file 2: Fig. S5A, Additional file 1: Table S5). Together, these observations strongly support the importance of RFX2 for neuron development and ciliogenesis.

The STRT RNA-seq expression level of the *RFX2* gene was comparable between WT and RFX2 -/- (Additional file 2: Fig. S4D), which should be the case, because the CRISPR/Cas9 gene editing we performed, did not affect the 5’ end of *RFX2* gene (Fig. 3A, Additional file 2: Fig. S1A-D). To confirm reduced (or absent) RFX2 TF function in the RFX2 -/- background, we examined the enrichment of TF binding site motifs closely associated with significantly downregulated TFEs at each time point. RFX2 binding motifs (X-boxes) were most significantly enriched at d4 and d5 (Additional file 2: Fig. S5B), indicating that RFX2 target genes were downregulated in the RFX2 -/- background. We further discovered that binding sites for the Atf, Jun, and Fos families of TFs were significantly enriched at d2 and d3 (Fig. 6D). These TFs are members of the AP-1 transcriptional complex that is activated by WNT signaling [28, 49]. In turn, this suggests that RFX2 contributes to the activation of the WNT signaling pathway.

### WNT signaling is essential to promote neuron differentiation

Primary cilia act as transducers of signaling pathways important for cortex development and axon pathfinding [50], neuron migration [30] and neuronal circuitry formation [32]. Thus, malfunction of ciliary signaling may lead to ciliopathies with brain phenotypes, to neurodevelopmental disorders or brain conditions [11, 26, 51]. Neural and neuron differentiation results from crosstalk of multiple (ciliary) signaling pathways [13], where WNT signaling was shown to play a highly relevant role [52], for example in neural stem cell differentiation [21]. Our STRT RNA-seq data revealed LUHMES RFX2 -/- neurons to be delayed in differentiation, whereby a number of downregulated genes in RFX2 -/- appeared to be targets of TF families (Atf, Jun, Fos) that are involved in non-canonical WNT signaling (Fig. 6D).

**Fig. 7:**
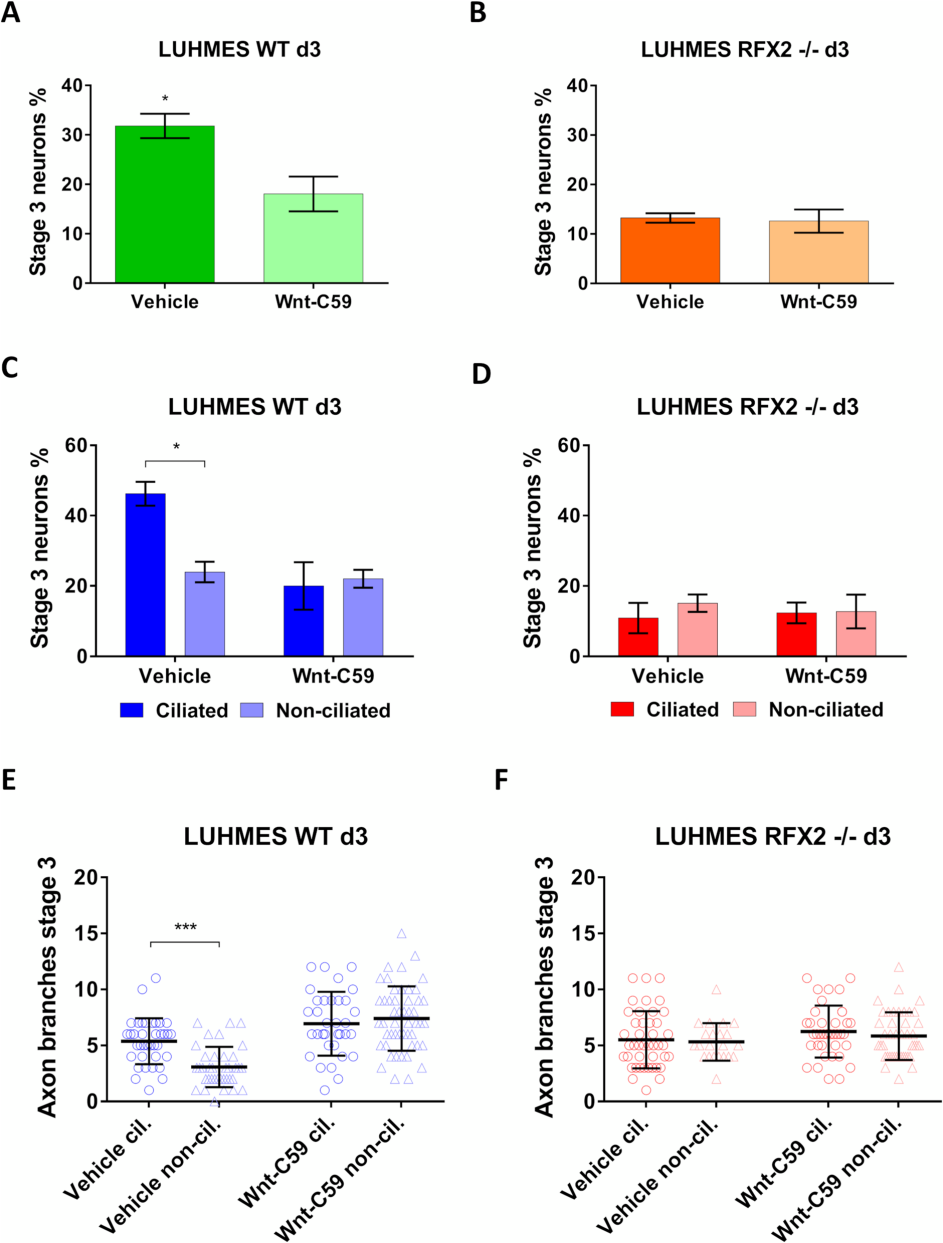
WNT signaling is essential for promoting human LUHMES neuron differentiation. **A**-**B** Inhibiting WNT signaling by treating LUHMES neurons with the antagonist Wnt-C59 negatively affects axon outgrowth (how efficiently neurons reach differentiation stage 3) in (**A**) WT (vehicle *n* = 190; Wnt-C59 *n* = 301) but not in (**B**) an RFX2 -/- background (vehicle *n* = 214; Wnt-C59 *n* = 242). Control (vehicle): the same treatment, but without antagonist. **C**-**D** WT ciliated neurons (*n* = 99) reach differentiation stage 3 more efficiently than non-ciliated neurons (*n* = 91) in the control experiment (vehicle), while no difference was observed between ciliated (*n* = 129) and non-ciliated neurons (*n* = 172) after treatment with the antagonist Wnt-C59 (**C**). No differences were observed in RFX2 -/- neurons (**D**) under the same experimental conditions (ciliated (*n* = 114) versus non-ciliated (*n* = 100); with (*n* = 129) versus without (*n* = 113) antagonist Wnt-C59). **E**–**F** Wnt-C59 treatment deregulates axon branching. In the control experiment (vehicle), axon branching is promoted in WT ciliated neurons (n = 37) as compared to non-ciliated neurons (*n* = 38). Upon treatment with the antagonist Wnt-C59 this difference is alleviated (ciliated *n* = 34; non-ciliated n = 50) (**E**). In RFX2 -/- neurons with altered cilia there are no differences in axon branching between ciliated (*n* = 43) and non-ciliated neurons (*n* = 22) in the control experiment (vehicle); likewise, for the treatment with the antagonist Wnt-C59 (ciliated *n* = 38; non-ciliated *n* = 43) (**F**). Mean values are shown ± s.e.m. (**A**-**D**) and ± s.d. (**E**–**F**). The results are from two independent experiments with a minimum of three technical replicates each. We conducted an unpaired two-sided t-test (95% confidence level) (**A**-**B**), and regular two-way ANOVA analyses (not repeated measures) with multiple comparisons (Bonferroni’s test) between conditions (**C**-**F**). *p < 0.05; ***p < 0.0005

To determine whether ciliary WNT signaling has an impact on LUHMES neuron differentiation, we used a pathway inhibitor (Wnt-C59) [53]. After controlling that Wnt-C59 treatment did not cause any difference in ciliation percentage (data not shown), we examined its impact on the two different phenotypes we previously uncovered in differentiating neurons (at d3): axon outgrowth (how efficiently neurons reach differentiation stage 3) and subsequent axon branching/arborization. In WT the axon marker TAU highlighted a significantly reduced number of stage 3 neurons upon treatment with Wnt-C59 (Fig. 7A), while no differences between control and Wnt-C59 treatment were observed in the RFX2 -/- background (Fig. 7B). When comparing ciliated to non-ciliated neurons in WT, we revealed a strong ciliary contribution to promoting axon outgrowth (Fig. 7C), which was abolished when WNT signaling was inhibited (Fig. 7C) or under any condition in RFX2 -/- neurons (Fig. 7D). We then used Phalloidin staining cytoskeletal F-actin to detect the shapes of entire neurons, including the number of axon branches/arborization. Again, WT ciliated neurons had more axon branches than non-ciliated neurons (Fig. 7E, Additional file 2: Fig. S2B), a difference that was abolished upon treatment with Wnt-C59, curiously though at a higher level of branching (Fig. 7E, Additional file 2: Fig. S2B), or under any condition in RFX2 -/- neurons (Fig. 7F, Additional file 2: Fig. S2B).

We also tested by qRT-PCR another common ciliary signaling pathway (SHH) (Additional file 2: Fig. S6A) and the expression of its canonical target genes during neuron differentiation (d3) in WT and RFX2 -/- when treated with either the SHH pathway activator Smoothened agonist (SAG) or the inhibitor cyclopamine. Consistent with a poor activation of the canonical SHH pathway during neuron differentiation [31, 54], we did not detect any apparent differences between the two genotypes (WT vs RFX2 -/-). Moreover, time course expression patterns of SHH target genes from our STRT RNA-seq data revealed that *GLI1* expression decreased when LUHMES neurons become post-mitotic (d1), *HHIP* was very poorly expressed overall, while *PTCH1/2* (negative regulators of canonical SHH pathway activation) were steadily expressed (Additional file 2: Fig. S6B).

Taken together, our results indicate a direct involvement of WNT signaling in different aspects of neuron differentiation, like axon outgrowth and subsequent branching. Disrupting WNT signaling by Wnt-C59 caused axon outgrowth and branching phenotypes that mimicked the outgrowth and branching phenotypes observed in control (vehicle) RFX2 -/- neurons, further strengthening the connection between WNT signaling and primary cilia for promoting neuron differentiation.

### Primary cilia regulate the subcellular localization of β-catenin, the main mediator of WNT signaling

How might primary cilia be involved in transducing WNT signaling? β-catenin is the main mediator for the activation of canonical WNT signaling and it is well expressed in both LUHMES WT and RFX2 -/- neurons (Additional file 2: Fig. S4D). β-catenin can be active or inactive: the phosphorylated form is degraded by a multiprotein destruction complex in the cytoplasm; the non-phosphorylated, active form is stabilized and translocates into the nucleus to affect canonical WNT target genes expression [55] (Additional file 2: Fig. S7). Cilia have been described as crucial regulators of the subcellular localization, and therefore activity, of β-catenin [56, 57].

**Fig. 8:**
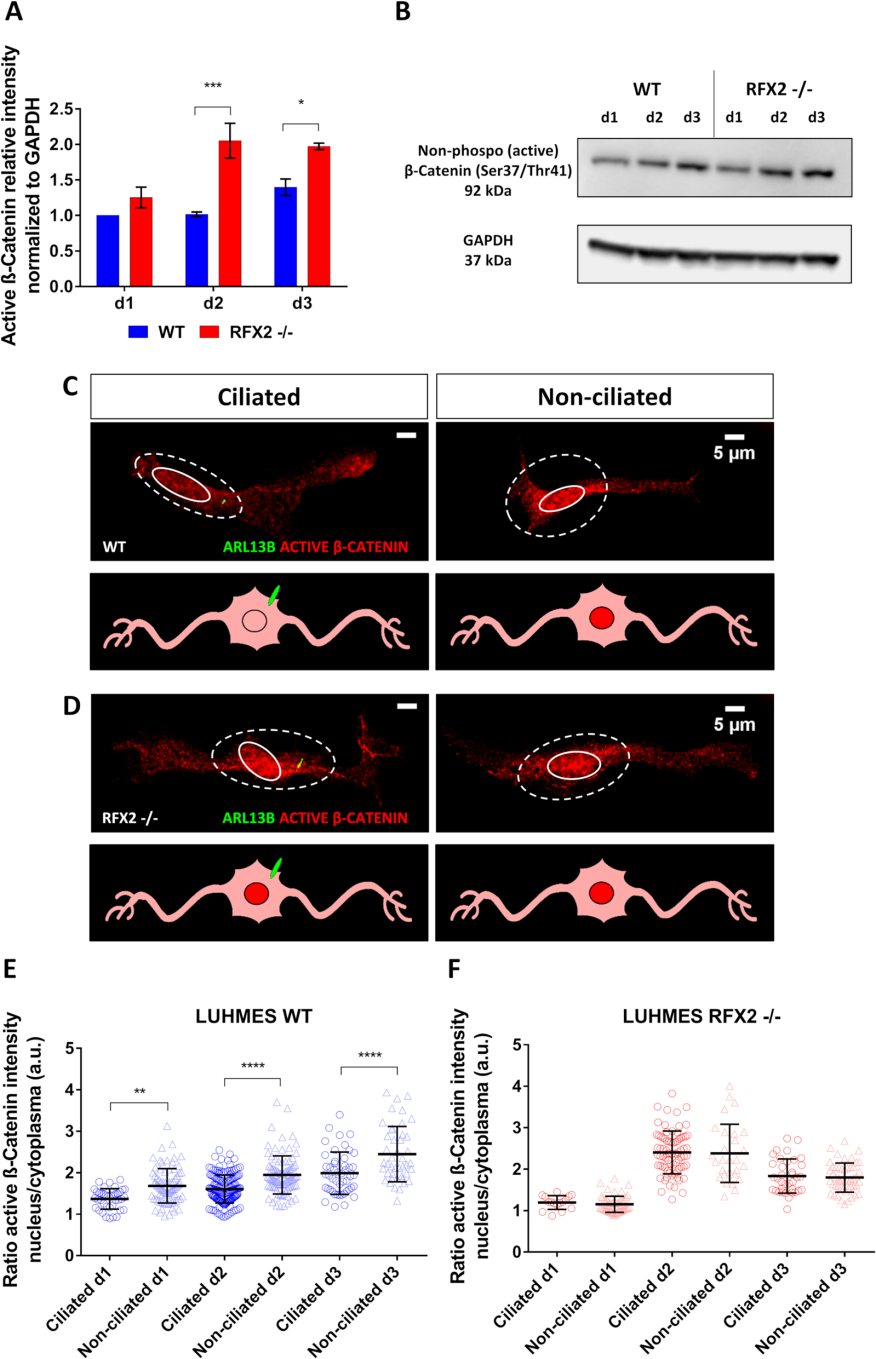
The intracellular localization of β-catenin, the major WNT signaling pathway mediator, is regulated by primary cilia. **A**-**B** The active (non-phosphorylated) form of β-catenin is significantly upregulated in an RFX2 -/- background as compared to WT neurons. This upregulation is particularly strong on d2 and d3 of neuron differentiation, the two time points with the highest percentage of ciliation (cf. Fig. 2A). Quantification of expression values (**A**) was carried out from western blot analysis (**B**) relative to the housekeeping protein GAPDH (loading control). **C**-**D** Active β-catenin immunocytochemistry staining of WT and RFX2 -/- neurons highlights differences between genotypes in intracellular protein localization: note the more prominent nuclear localization in the RFX2 -/- background and in non-ciliated WT neurons as compared to ciliated WT neurons. Circles define nuclei and dashed lines define the cell body; the ciliary marker is ARL13B (green). **E**–**F** Quantification of active β-catenin localization based on the ratio of immunofluorescence intensity between the cytoplasm and the nucleus on d1, d2 and d3 of LUHMES neuron differentiation. In WT, ciliated neurons (*n* = 39–167) more strongly promote β-catenin degradation in the cytoplasm, resulting in diminished translocation to the nucleus of the active form (lower ratio), as compared to non-ciliated neurons (*n* = 44–95) (higher ratio) (**E**). In an RFX2 -/- background, ciliated (*n* = 16–77) and non-ciliated neurons (*n* = 28–57) show no differences in regulating β-catenin intracellular localization (equal ratios) (**F**). Mean values are shown ± s.e.m. (normalized to GAPDH) (**A**) and ± s.d. (**E**–**F**). The results are from two independent experiments with a minimum of two technical replicates each. We conducted regular two-way ANOVA analyses (not repeated measures) with multiple comparisons (Bonferroni’s test) between groups. *p < 0.05; **p < 0.005; ***p < 0.0005; ****p < 0.0001

To determine how the cilia of LUHMES neurons influence β-catenin turnover and consequently WNT signaling, we quantified the expression of the active (non-phospho) form of β-catenin by Western blot. Given the ciliation pattern of LUHMES neurons during the early neuron differentiation time window (cf. Figure 2A), we analyzed active β-catenin during d1-d3 in both WT and RFX2 -/- backgrounds. Different from the overall β-catenin expression (Additional file 2: Fig. S4D), we found a significant upregulation of the active form in RFX2 -/- on d2 and d3 (Fig. 8A, 8B, Additional file 3: Fig. S1B). Differentiating neurons at d1 did not result in significant differences between WT and RFX2 -/-, which may be due to the still low percentage of ciliation (about 30%) right after release from precursor cell proliferation into neuron differentiation, as compared to the following differentiation days (about 50% ciliation for d2 and 70% for d3) (cf. Fig. 2A).

To uncover the involvement of cilia in regulating the balance of cellular β-catenin localization, we performed immunocytochemistry to visualize where in differentiating neurons active β-catenin localizes. We compared fluorescence intensities (A.U.) detected in the nucleus and the surrounding cytoplasm and found that non-ciliated WT neurons were less efficient than ciliated neurons in inhibiting the nuclear activation of β-catenin (Fig. 8C, E). While no β-catenin localization differences were detected in RFX2 -/- neurons between populations with altered cilia and non-ciliated populations (Fig. 8D, F). Finally, consistent with the detection of bands on Western blots (Fig. 8A, B, Additional file 3: Fig. S1B), also immunocytochemistry revealed a stronger nuclear presence of active β-catenin in RFX2 -/- as compared to WT neurons (Fig. 8D, F).

These findings suggest that cilia and ciliary signaling pathways do not regulate overall β-catenin protein levels but rather the subcellular turnover and localization of β-catenin. Furthermore, differentiation-delayed LUHMES RFX2 -/- neurons showed a higher activation of the β-catenin-mediated canonical WNT pathway, whereas LUHMES WT neurons appeared more efficient in switching to non-canonical WNT activation promoting neuron differentiation [21].

### WNT signaling is involved in the organization of the cyto-architecture

Neuron differentiation requires cytoskeletal rearrangements to form the outgrowth and subsequent branching of neurites [58], and to generate synapses. Downstream effects of non-canonical WNT signaling can lead to modifications of the cyto-architectural organization [59]. To test whether the differentiation changes observed in LUHMES RFX2 -/- neurons were due to a disrupted balance between canonical and non-canonical routes of the WNT signaling pathway with a consequent impairment of cytoskeletal structure, we treated neurons with a pathway inhibitor (Wnt-C59) or with a canonical pathway activator (Wnt3a) [60]. The expression of WNT pathway output genes implicated in cyto-architectural functions [61, 62] was subsequently assessed by qRT-PCR.

**Fig. 9:**
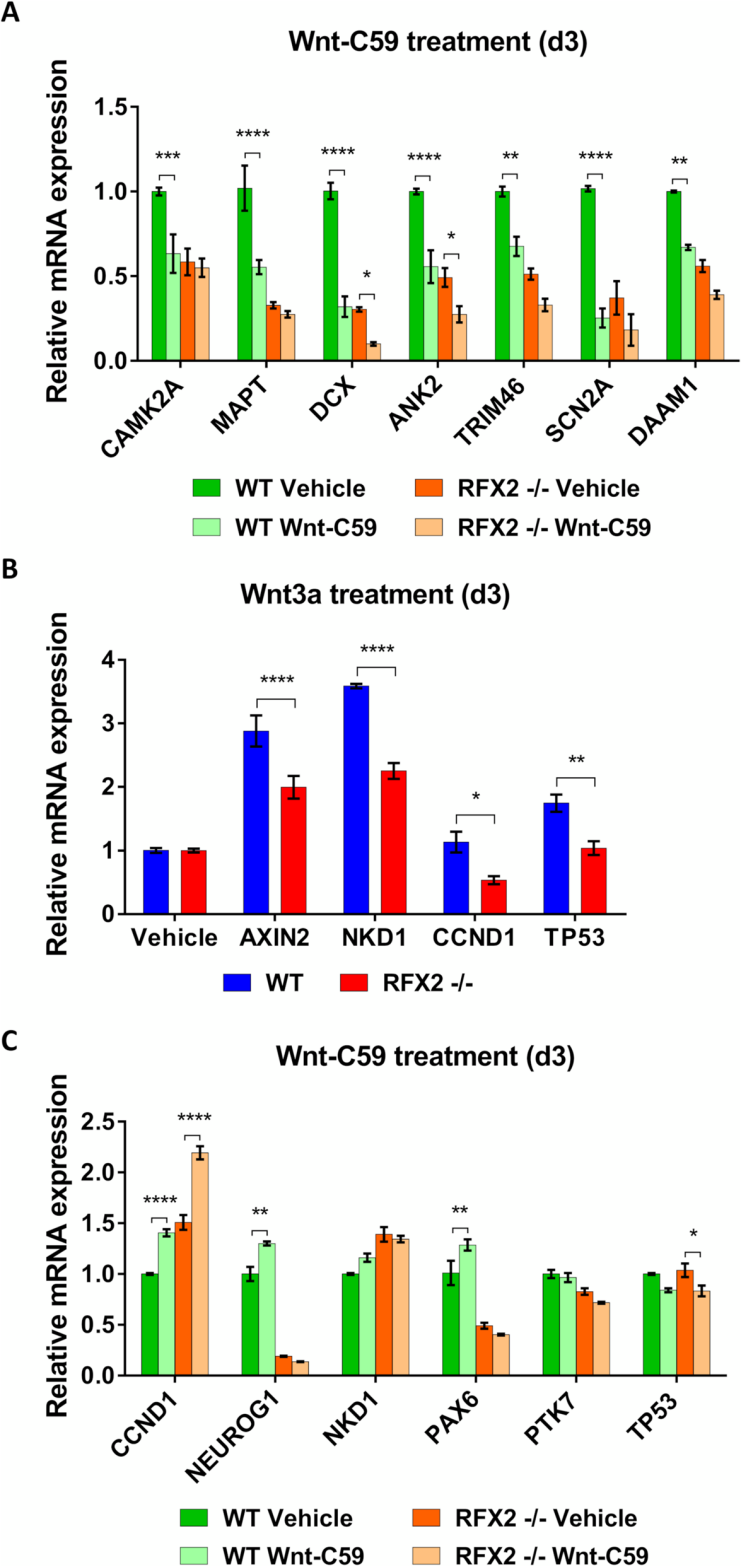
The ciliary WNT signaling pathway regulates the expression of cyto-architectural remodeling genes during neuron differentiation. qRT-PCR analysis of the expression of WNT signaling pathway output genes following pathway inhibition (antagonist Wnt-C59) or activation (agonist Wnt-3a) reveals clear gene expression differences in LUHMES WT neurons as compared to RFX2 -/- neurons with an altered primary cilium. **A** WT neurons treated with Wnt-C59 show strong downregulation of genes implicated in cyto-architectural organization, including the non-canonical WNT signaling pathway output genes *CAMK2A* and *DAAM1*: expression levels are very similar to the basal expression levels of untreated RFX2 -/- neurons (vehicle). **B** Treatment with a WNT activator (Wnt-3a) results in stronger upregulation of canonical output genes in WT neurons as compared to in the RFX2 -/- background. **C** qRT-PCR analysis of the expression of a set of canonical WNT signaling pathway output genes (Note: *PTK7* is also as non-canonical target gene) following pathway inhibition (antagonist Wnt-C59): for this set of output genes WT neurons treated with Wnt-C59 do not show downregulation of gene expression and their expression levels are very similar to the basal expression levels of untreated (vehicle) RFX2 -/- neurons with an altered primary cilium (exceptions: neuron differentiation markers *NEUROG1* and *PAX6*). These results point toward an involvement of the non-canonical WNT signaling pathway in neuron differentiation to promote cytoskeletal rearrangement and remodeling. Mean values are shown ± s.e.m. (normalized to GAPDH; not shown). The results are from a minimum of three independent experiments with two technical replicates each. We conducted regular two-way ANOVA analyses (not repeated measures) with multiple comparisons (Bonferroni’s test) between groups. *p < 0.05; **p < 0.005; ***p < 0.0005; ****p < 0.0001

Treatment with a pathway inhibitor (Wnt-C59) revealed clear differences on how these WNT output genes responded: they were strongly downregulated in WT and only reached similarly basal expression levels in RFX2 -/- (Fig. 9A). Aside from their structural functions, these genes are also widely known as axon markers (*MAPT, TRIM46*; [63, 64], non-canonical WNT pathway output genes (*CAMK2A, DAAM1*, [62, 65] and as candidate genes for neurodevelopmental disorders (*CAMK2A, ANK2, DCX, SCN2A*, [66–69], highlighting their importance for cell functionality. Canonical WNT output genes were also tested following Wnt-C59 inhibition, but typically no significant differences in expression levels were detected (Fig. 9C), further strengthening the importance for neuron differentiation of non-canonical WNT signaling (over the canonical route). Finally, the activation of the canonical WNT pathway with the activator Wnt3a showed a stronger upregulation of canonical target genes in WT, but not in the RFX2 -/- background (Fig. 9B), where altered cilia may prove less efficient in detecting and transducing extracellular cues.

These results describe molecularly how neuron differentiation changes and/or delays (axon outgrowth and subsequent branching), as observed in RFX2 -/- neurons, may arise in the absence of a fully functional signal transduction hub like the cilium: a deregulation of WNT signaling leads to impairment and defects in the cytoskeletal rearrangement capacities and consequently to impaired neuron differentiation.

## Discussion

We investigated how cilia and ciliary signaling affect the development of the nervous system, using LUHMES, an established and validated human neuronal cell model [26, 70], where we can differentiate proliferating neuronal precursor cells into fully mature, functional neurons within one week. We used wild-type (WT), a CRISPR/Cas9 designed mutant for the ciliogenic transcription factor (TF) RFX2, and siRNA knockdowns (KD) of the essential ciliary genes *IFT88* and *IFT172*.

We could show that functional cilia and ciliary signaling are required to promote neuron maturation during an early differentiation time window: cilia promote axon outgrowth and regulate subsequent axon branching/arborization. These crucial aspects of differentiation were deregulated in neurons with mutated and altered cilia (RFX2 -/-, *IFT88* KD, *IFT172* KD). Microscopy and molecular data from STRT RNA-seq analyses demonstrated a delayed differentiation of RFX2 -/- neurons as compared to WT. When testing a highly relevant (ciliary) signaling pathway for neuron differentiation, WNT, we found that cilia play a key role in regulating the cellular turnover of β-catenin, the main mediator of WNT signaling, significantly decreasing its nuclear translocation and consequently, the canonical activation of the pathway.

In our assays the non-synchronized populations of post-mitotic, differentiating LUHMES neurons consisted of both ciliated and non-ciliated neurons. Our complete differentiation time course experiments from precursor to mature neuron revealed a clear pattern: the presence of cilia steadily increased and peaked at d3 of differentiation (around 70% ciliation) before decreasing during later differentiation stages (d4-d6). Cilia are dynamic organelles during the cell cycle [71], during neuron differentiation both in vivo and in vitro [24, 31, 42] and during the differentiation of non-neuronal cell types [57, 72]. Similarly, ciliation in our post-mitotic, differentiating LUHMES neurons is dynamic and transient, suggesting a ciliary time window that appears to “open” *after* newborn neurons undergo the first centrosome-associated differentiation steps (polarization, growth cone development, initiation of neurites) [36, 37] and to “close” (disassembly of cilia) *before* the emergence of large numbers of fully functional synapses as signaling hubs during the later neuron differentiation and maturation stages [36]. This ciliary time window appears to be centered around differentiation stages 2 and 3 (breaking the bipolar symmetry of neurites and axon outgrowth). Importantly, we found that ciliation conferred an outgrowth advantage: neurons with detectable cilia progressed more efficiently than non-ciliated neurons to stage 3 of differentiation. We note that our in vitro experimental setup did not allow for including the analysis of neuron migration, an important, and in parts concurrent, aspect of neuron differentiation and maturation.

To study whether and how ciliary alterations might affect neuron differentiation, we mutated the ciliogenic TF gene *RFX2* and knocked down the expression of two essential ciliary genes, *IFT88* and *IFT172* [73]. In human, there are eight *RFX* genes [8]. LUHMES cells and neurons prominently express several RFX family members with specific, but also potentially redundant roles (RFX1-3, RFX5), where RFX5 has so far not been associated with ciliogenesis [6]. *RFX2* has also been connected to spermatogenesis [74–76] but, within the nervous system, has only been connected to ciliogenesis and in LUHMES is highly expressed at d0-d2 of differentiation (our transcriptomics data). We found ciliary genes [43], including relevant ciliary signal transduction genes, significantly differentially expressed between WT and RFX2 -/- neurons at d3, d4, d5 of differentiation, and RFX2 -/- cilia were not truncated but significantly longer than WT cilia. This was surprising considering previous studies where reduced *RFX2* expression caused shorter cilia, albeit in different organisms (Xenopus and Zebrafish) and by using transient downregulation of gene expression [9, 10], as compared to the stable gene knockout from our work. Elongated cilia suggest a potential role of the RFX2 TF in negatively regulating aspects of ciliogenesis at the onset of neuron differentiation, while RFX1 and RFX3, which are upregulated later, may be involved in regulating ciliogenesis after d1, when ciliary signaling promotes the differentiation process.

Our research aimed to uncover cellular and molecular mechanisms (when, where and how) by which cilia promote neuron development and differentiation. Our findings in WT that ciliated neurons more efficiently than non-ciliated neurons (i) reached differentiation stage 3 (break of symmetry, axon outgrowth) and (ii) generated significantly more branches on emerging axons, strengthens the concept that cilia are involved in critical, axon-associated aspects of neuron differentiation. By contrast, altered cilia of RFX2 -/- and *IFT88*, *IFT172* KD neurons did not provide a differentiation advantage: fewer ciliated neurons reached stage 3 of differentiation while for axon branching the regulation appeared unrelated. Interestingly and differently from WT and *IFT88*, *IFT172* KDs (with significantly shorter cilia), RFX2 -/- axons from non-ciliated neurons were as highly branched as neurons with altered cilia, again implicating RFX2 in a potentially negative regulatory role regarding axon branching, which in turn may later facilitate axon pruning. Our results suggest that although part of the same overall neurodevelopmental process, mechanisms for axon outgrowth and subsequent axon branching may (in parts) be independent from each other, possibly requiring different types and different timing of cytoskeletal remodeling. The specificity of the ciliary impact on neuron (axon) differentiation is further underscored by the fact that populations of WT and RFX2 -/- neuronal precursor cells and neurons did not display any relevant differences in proliferation rates nor in neuron types (bipolar neurons dominated in populations of both genotypes).

We complemented our cellular observations with analyzing the STRT RNA-seq transcriptomes of both genotypes in time course experiments. RFX2 -/- neurons were delayed in differentiation as compared to WT neurons at the same time point. Ciliary alterations (like in RFX2 -/-) therefore seem to produce a relatively mild phenotype (as it frequently is the case in ciliopathies), where neurons differentiate like in WT, but with a different timing, which may disrupt critical spatiotemporal aspects of brain formation. Gene expression profiles from transcriptomics were grouped into three macro-clusters, whereby we particularly focused on Cluster 3 because the GO analysis of sets of downregulated genes in RFX2 -/- neurons showed strong enrichments of functional characteristics related to the nervous system, neuron and axon development, explaining the RFX2 -/- delay in neuron differentiation at the molecular level. Moreover, sequence motifs associated with downregulated genes were strongly enriched for binding sites of TFs prominently involved in WNT signaling, a cilia-associated pathway well-known for regulating neuron development [18, 52]. For comparison, to promote neuron differentiation the SHH signaling pathway switches from canonical to non-canonical activation in post-mitotic neurons [31, 54], yet the non-canonical SHH pathway does not require primary cilia to be transduced [77].

Different types of small molecules are available to modulate WNT signaling at different levels of the pathway [78]. LUHMES neurons are an in vitro model where the culture conditions do not fully reflect an in vivo extracellular environment with exogenous WNT ligands. As such, the WNT pathway in LUHMES is mainly regulated by endogenous WNT signals produced in the cell. Furthermore, both the canonical and non-canonical pathway routes [13] may simultaneously be operative. Therefore, we treated differentiating LUHMES neurons with the Wnt-C59 antagonist [53] to inhibit the pathway in full, preventing secretion and subsequent activation of all endogenous WNT signals, regardless of their pathway affinity. In WT neurons this resulted in a significant reduction of axon outgrowth (stage 3 neurons) irrespective of the presence or absence of cilia, and increased and deregulated axon branching, a phenotype also observed in the RFX2 -/- background. RFX2 -/- neurons treated with Wnt-C59 or vehicle (control) showed no differences between ciliated and non-ciliated neurons in both conditions, with a decreased percentage of stage 3 neurons as compared to WT and a similar upregulation of axon branching in non-ciliated neurons in both experimental conditions. These results clearly demonstrate that WNT signaling affects neuron differentiation and axon outgrowth, potentially coordinating the formation of necessary *versus* eventually unnecessary branches, a key aspect of subsequent neuron interconnectivity and circuitry.

To elucidate where and how cilia regulate WNT signaling, we determined the cellular localization of the active form of β-catenin, noticing in WT a significant reduction of the ratio nucleus/cytoplasm in the presence of cilia, while in RFX2 -/- with altered cilia and thus, apparent modulation deficits, ciliated and non-ciliated neurons displayed similarly high ratios nucleus/cytoplasm. These results reveal that cilia are important for controlling (and reducing) the nuclear translocation of β-catenin rather than mediating its cytoplasmic degradation. Since β-catenin is the main mediator of canonical WNT signaling, this indicates that in (LUHMES) neurons it is functional cilia that reduce the activation of canonical WNT signaling, to favor the non-canonical route for promoting the rearrangement of cytoskeletal structures required to develop and grow neurites and branches during early neuron differentiation [21]. Our results are consistent with previous observations [79], although the precise role of cilia in canonical WNT signaling pathway regulation is still controversial [80]. Further, inhibition with Wnt-C59 also led to a strong downregulation of key genes involved in cytoskeletal remodeling [81], many of which were similarly downregulated to basal expression in RFX2 -/-. In addition, by exposing LUHMES neurons to an exogenous canonical WNT signaling pathway agonist (Wnt3a) [82] we found that WT neurons (with WT cilia) were better equipped than RFX2 -/- neurons (with altered cilia) to transduce environmental stimuli and, as in the case of Wnt3a treatment, to upregulate the expression of canonical WNT signaling target genes. In summary, ciliary regulation of WNT signaling and its output proves essential to drive neuron differentiation by translating signaling events into anatomical changes necessary to shape the emerging axon and its branching pattern. Testing further combinations of pathway activator/inhibitor molecules will in the future be needed to dissect and resolve the when and where of ciliary regulation of WNT signaling output more precisely.

Based on the pattern of ciliation in LUHMES neurons (steadily increasing and peaking around d3 of differentiation before decreasing again; particularly prominent at differentiation stages 2 and 3), we propose the following working model, integrating previous and our current results. The centrosome functioning as MTOC leads the initiation of neuron differentiation, driving precursor cell polarization and determining the axon formation site [36, 37]. Ciliogenesis ensues and thereby informative ciliary signaling becomes crucially important during an early differentiation time window to promote axon outgrowth (breaking the bipolar symmetry during differentiation stages 2 and 3), and subsequent axon branching and arborization. Later, during differentiation stages 4 and 5, ciliation declines and the establishment of many functional synapses (across a now much larger anatomical space) take over relevant signaling tasks for completing neuron differentiation and maturation [28]. We note that our working model does not consider neuron migration, a developmental process not tested, but which in vivo partially overlaps with the anatomical differentiation processes described here (neuron migration has also been shown to be subject to ciliary regulation – see below). Mechanistically, the ciliary impact on neuron differentiation is executed by tipping the balance from canonical to non-canonical WNT signaling, decreasing the nuclear translocation of β-catenin and, therefore, initiating cytoskeletal rearrangements to promote and sustain neuron differentiation.

Our previous [26] and current work in human LUHMES neurons on the ciliary impact on axon outgrowth and subsequent branching compares well with complementary work in other systems. Cilia and ciliary signaling were found to promote axonal growth cone architecture and axon tract development through the regulation of the PI3K/AKT signaling pathway, as revealed by using mice mutants for ciliary ARL13B [32]. Cilia and ciliary signaling proved essential for dendrite outgrowth and refinement as well as for the initiation of synapse formation [33, 83]. Stoufflet and colleagues (2020) described cilia as the crucial organelle for driving in vivo saltatory migration of neurons in the mouse brain through the cAMP/PKA pathway, being cyclically extended in the leading process (future dendrite) of migration. Whereby, disruption of neuronal ciliogenesis can lead to defective neuron migration and inactivation of non-canonical WNT signaling in association with aberrant cytoskeletal remodeling [59, 84].

Our work expands on previous studies as we provide in a human neuronal cell model, LUHMES, detailed and complete, cellular and molecular time course analyses of neuron development from proliferating neuronal precursor cell to differentiating and fully differentiated, mature neuron. In LUHMES it is possible to precisely analyze the function of cilia and ciliary signaling at every single step of neuron differentiation. Moreover, we have elucidated a molecular mechanism underlying the ciliary regulation of cytoskeletal rearrangements necessary to promote neuron differentiation through the activation of non-canonical WNT signaling. It is highly likely that WNT signaling cross-talks with other relevant ciliary signaling pathways like PI3K/AKT and cAMP/PKA [85, 86]. Thus, to fully understand the ciliary regulation of neuron differentiation, investigating other (interacting) ciliary signaling pathways, on top of our discovery of WNT signaling, will likely be required.

## Conclusions

Dysregulation of ciliary activity has increasingly been found to be implicated in neurodevelopmental conditions and disorders [11, 26, 87]. We hypothesize an early differentiation time window where cilia and ciliary signaling drive the anatomical transition from proliferating precursor to differentiated neuron, by promoting neuron migration and axon development needed for proper brain formation. We propose that ciliary malfunctions result in an inability to properly translate signaling events into anatomical changes, which can lead to impaired neuron migration to eventual brain destinations or to anatomical deficits, eventually causing localized circuitry defects and increasing the risk of the onset of certain neurodevelopmental conditions and disorders like autism, schizophrenia and dyslexia [7, 26, 88].

### Potential limitations of our study

LUHMES represents a simplified in vitro system comprised of a single type of neuron, lacking the support of and interactions with glial and endothelial cells that occur in vivo. In regular cell culture setups LUHMES affords very limited possibilities for investigating cell and neuron migration, a very important “partner” in neuron differentiation. While future 3D brain organoid setups may be needed to fully understand and explore the ciliary contributions to brain formation, several in vitro approaches have already been established as valuable tools to study neuron migration at the cellular and molecular level and these can be adjusted for being applied in LUHMES neuron cultures as well [89]. However, a simplified system may represent an advantage when it comes to isolate, clearly separate, and analyze specific neuron differentiation steps. LUHMES neurons make possible to analyze the complete neuron differentiation process in unprecedented detail at every single step. A future system upgrade will be the ability to continuously follow individual fluorescently marked cells or neurons over time by using live cell imaging approaches (transient or permanent transgenesis) to uncover in more minute detail the flow (rather than discrete steps) of cilia-promoted neuron differentiation. Live visualization of individual neurons will make possible the accurate molecular analyses by single cell RNA-sequencing through the ability to selectively pick ciliated and non-ciliated cells and neurons from the same culture. LUHMES is a hard-to-transfect cell line, though reasonably good results have been obtained using novel electroporation protocols [90, 91], making LUHMES suitable for genome editing by CRISPR/Cas9 at a larger scale.

## Methods

### Cell culture and growth conditions

Lund human mesencephalic (LUHMES) cells, a v-myc immortalized neuronal precursor cell line, was obtained from the ATCC (https://www.atcc.org/products/crl-2927). LUHMES cells were cultured in a standard incubator (37 °C, 5% CO_2_) as previously described [26, 42, 70]. LUHMES cells were grown in DMEM/F-12 Ham growth medium (Sigma-Aldrich D6421) supplemented with L-glutamine solution (Sigma-Aldrich G7513; 2.5 mM), N-2 supplement (Gibco 17502048; 1 ×) and human heat stable basic Fibroblast Growth Factor (bFGF) (Thermo Fisher Scientific PHG0369; 20 ng/ml) in vessels pre-coated with poly-L-ornithine hydrobromide (Sigma-Aldrich P3655; 50 μg/ml) and fibronectin from human plasma (Sigma-Aldrich F1056; 1 μg/ml). For some experiments, a higher concentration of ornithine (100 μg/ml), fibronectin (10 μg/ml) and bFGF (40 ng/ml) was used to improve cell adhesion and propagation. To differentiate LUHMES neuronal precursor cells into post-mitotic neurons, bFGF in the growth medium was replaced with tetracycline hydrochloride (Sigma-Aldrich T7660; 1 μg/ml) to terminate v-myc transgene expression.

### Immunocytochemistry and fluorescence intensity quantification

Immunocytochemistry was performed as previously described [38, 42]. 7.5 × 10^4^ LUHMES cells were seeded 24 h prior to differentiation into pre-coated 35 mm dishes (Corning 353001) containing round borosilicate cover glasses (VWR 631-0150P), pre-treated with hydrochloric acid fuming 37%, rinsed with dH_2_O and absolute ethanol and finally stored in 70% ethanol. Cells were fixed in 2% paraformaldehyde (PFA) solution (Invitrogen FB002) for 5 min in the incubator and permeabilized with 0.2% Triton X-100 in PBS for 12 min at room temperature (RT). The glass cover slips were then incubated with blocking buffer (2% bovine serum albumin and 0.1% Tween 20 in PBS) for 30 min at RT, prior to primary antibody incubation overnight at 4 °C and secondary antibody incubation for 1 h at RT. Cells on glass cover slips were rinsed twice in PBS before being mounted on microscope slides (Thermo Fisher Scientific AA00000102E) using 4 μl of ProLong glass antifade mountant (Invitrogen P36982). Samples were then left in the dark on a flat surface for 24 h at RT to allow them to cure and reach the optimal refraction index.

All primary and secondary antibodies were diluted in blocking buffer and used under the following working conditions. Neuron differentiation markers were chicken anti-TAU (MAPT) (Abcam ab75714; 1:100), rabbit anti-TRIM46 (Human Protein Atlas HPA030389; 1:100), mouse anti-TUBB3 (TUJ1) (BioLegend 801213; 1:3000) and rabbit anti-PSD95 (Cell Signaling 2507; 1:100). Centrosome (centriole, basal body) and cilium markers were, respectively, mouse anti-PCNT (Abcam ab28144; 1:250) and rabbit anti-ARL13B (Proteintech 17711-1-AP; 1:10,000). Mouse anti-active-β-catenin (Sigma-Aldrich 05–665; 1:500) was used to detect the main cellular mediator of WNT signaling. Secondary antibodies were conjugated with Alexa Fluor dyes 488, 555, 647 (Invitrogen) and used at a 1:600 dilution. Alexa Fluor 647 Phalloidin staining (Invitrogen A30107; 1 ×) was coupled with secondary antibody incubation to visualize F-actin. Nuclei were stained for a few seconds with the Hoechst dye 33342 (Invitrogen H3570; 50 μg/ml in PBS). The intensity of active-β-catenin fluorescence was measured as the mean value in arbitrary units (A.U.) and quantified using Fiji software [92]: we divided the value obtained from each nucleus (localized through Hoechst staining) by the average of three anatomically separate cytoplasmic measurements to minimize measurement errors.

### Microscopy and image acquisition

Microscopy work was performed at the Live Cell Imaging Core facility/Nikon Center of Excellence, at the Karolinska Institute (https://ki.se/en/bionut/welcome-to-the-lci-core-facility). For immunocytochemistry we used a Nikon Ti-E inverted spinning disk CREST v3 confocal microscope with a fully motorized piezo stage for 3D z-stacking and a Plan Apo λ 60 × NA 1.40 oil immersion objective, matched to the refractive index of the sample mounting medium and cover glasses. Images were acquired with a Kinetix sCMOS camera (> 95% quantum efficiency, 6.5 μm pixel size, 2720 × 2720 pixels field of view).

Measurements of differentiating axons: Neurites were marked with anti-TAU or anti-TRIM46 as axon markers. The length and diameter of neurites were measured using the line tool of the Fiji software [92]. Neurite length was measured as the trajectory starting at the center point of the nucleus (stained with Hoechst dye 33342) and reaching the tip of the neurite. Only neurons with the main neurite (emerging axon) being at least 1.5 times longer than the opposite neurite were included in the differentiation stage 3 group of neurons: after the break of bipolar symmetry.

Cilia length measurements: Cilia in LUHMES emanate from the neuronal cell body [26]. We marked ciliary basal bodies with anti-PCNT and the entire ciliary shaft with anti-ARL13B. We used an optimized microscopy z-stack imaging setup (0.3 μm slice thickness at Nyquist sampling), in which cilia were freely “floating” in the mounting medium: cilia were physically not obstructed in any of the XYZ axes by neither the glass slide nor by the cover glass [42]. The length of individual cilia identified by anti-ARL13B fluorescence was then measured base-to-tip using the line tool of the Fiji software [92] before applying the Pythagorean theorem (PyT) method previously described and validated [93]. This method allows to measure cilia length regardless of their 3D orientation in an unbiased high-throughput way.

### CRISPR/Cas9 mutation of the RFX2 gene in LUHMES cells

We generated an RFX2 (-/-) mutant LUHMES cell line by using CRISPR/Cas9-mediated non-homologous end joining. To minimize off-target effects the double-nicking strategy with the Cas9 D10A nickase mutant was used in combination with paired guide RNAs [94] (for sgRNA sequences see Additional file 1: Table S6). We double-checked for potential off-target effects by using software (https://cctop.cos.uni-heidelberg.de:8043), [95] that determines and analyzes the predicted number of sequence mismatches and their distance from the PAM sequence that would need to be tolerated by the Cas9 enzyme. The only gene with 0 mismatches with the designed sgRNAs was *RFX2*. All the other genes listed as off-target candidates had at least 4 mismatches, including in the PAM sequence “core region”. Annealed oligonucleotides covering the 20 nt guide sequences and an NGG PAM site were then cloned into the BbsI restriction site of the plasmid pSpCas9n(BB)-2A-GFP (pX461; Addgene plasmid #48140; gift from Feng Zhang). The resulting plasmid constructs were verified by Sanger-sequencing prior to use.

Approximately 1 × 10^6^ LUHMES cells were seeded into coated 10 cm petri dishes in complete medium (Sigma-Aldrich D6421). Transfection was carried out the following day at about 40% confluency. 7.5 μg of each sgRNA containing plasmid (15 μg in total) were transfected together with 24 μl lipofectamine (Invitrogen 18324012) in 5 ml fresh complete medium overnight. The following day fresh complete medium was re-supplied, and cells were allowed to recover for 48-72 h. For cell-enrichment, transfected LUHMES cells were FACS-sorted and GFP expressing cells were collected for further culture. Prior to sorting, cells were trypsinized and singled-out in PBS, followed by a passage through a wet cell strainer (mesh size 40 μm). For clone isolation approximately 1000 FACS-sorted GFP-positive cells were seeded per 10 cm dish in 8 ml complete medium and cultured for 10 days with one medium change. On day 10 single cell-derived colonies were picked up using a 100 μl pipette tip and transferred into a 96-well plate containing complete medium. Clones were sub-cultured continuously until the plate wells were fully covered. At this point replicas of isolated clones were generated and sub-cultured in a 24-well plate format. One replica was used for further culturing, while the other one was used to screen for mutations in the RFX2 gene. In parallel, LUHMES WT cells used as controls in all experimental procedures, followed a similar clonal selection scheme as the RFX2 -/- mutant clones.

For screening, cells were trypsinized and pelleted by centrifugation. Extraction of template genomic DNA was performed by using QuickExtract (Epicentre QE0905T). 2 μl of genomic DNA extract solution was used to PCR amplify the genomic locus of interest (*RFX2*) with an intronic primer pair. PCR-products were subsequently Sanger-sequenced for the identification of mutations in the *RFX2* gene. Location and sequence details of the CRISPR/Cas9-generated mutations in the *RFX2* gene are shown in Additional file 2: Fig. S1.

In summary, we generated a deletion-followed-by-frameshift mutation of *RFX2* upstream of its defining functional domain, the DNA binding domain (DBD). To independently confirm the successful mutagenesis of the *RFX2* gene, we designed primers for qRT-PCR complementary to consecutive exon sequences upstream of the DBD. As expected, and differently from the STRT RNA-seq data, qRT-PCR results showed a strong and significant downregulation of *RFX2* throughout the entire differentiation time course (Additional file 2: Fig. S1B), while no RFX2 protein at all was detectable on Western Blot (Fig. 3B, Additional file 3: Fig. S1A).

### Proliferation rate assay in different culture conditions

To assess cell proliferation rates in different conditions (proliferation versus differentiation), both wild type and RFX2 -/- LUHMES cells were seeded in parallel in pre-coated 6-well plates (Corning 353046) for growth overnight in regular proliferation medium until the next day (time point = 0 h). To assess growth in regular proliferation conditions, cells were grown for an additional 72 h until they became fully confluent. To assess growth under neuron differentiation conditions, proliferation medium was changed to differentiation medium at 24 h, and cells were kept in culture for 96 h in total. Images at different time points of culture were acquired using a bright-field microscope (Carl Zeiss, Axiovert 200 M) and then transferred into Fiji software [92] for manual cell counting.

### Western blot and protein expression quantification (RFX2, active β-catenin)

Wild type and RFX2 -/- LUHMES cells were cultured in a T75 flask until reaching 80% confluency. Cells were then washed twice with ice cold PBS and mechanically detached using a cell scraper (Sarstedt 83.3951) in RIPA lysis buffer (Millipore 20–188; 1 ×) 1% SDS solution after adding protease inhibitor (Roche 4693116001; 1×) and phosphatase inhibitor cocktails (Roche 4906845001; 1×). Cell samples were disrupted by vortexing, using a syringe, and clarified centrifugation at 14,000 × g for 15 min at 4 °C, followed by supernatant collection in a new Eppendorf tube. Protein concentration was determined using a BCA assay kit (Thermo Fisher Scientific 23227) and a microplate reader (Tecan M200 Infinite Pro). Protein samples were then mixed with LDS loading buffer (Invitrogen NP0007; 1 ×) and reducing agent (Invitrogen NP0009; 1 ×) and denatured at 70 °C for 10 min. 20 μg of protein sample and a reference ladder (Bio-Rad 1610374) were loaded in NuPage 4%-12%, Bis–Tris gel (Invitrogen NP0321PK2) and electrophoresis was carried out in MOPS SDS buffer (Invitrogen NP0001; 1 ×) at 200 V for 1 h. At the end of the gel run, transfer buffer was prepared (for 1 L: 14.4 g glycin, 3.03 g Tris Base, 200 ml methanol, up to volume with dH_2_O), the nitrocellulose membrane was activated in methanol for 5 min prior to the assembly of the transfer sandwich; the protein transfer was run at 100 V for 1 h at 4 °C. The membrane was retrieved from the sandwich and washed with PBS-T (0.1% Tween 20) for 5 min, before being blocked with 5% non-fat dry milk in PBS-T for 1 h and incubation with the primary antibody overnight at 4 °C on a shaker. The membrane was then washed three times in PBS-T and incubated with the secondary antibody for 1 h at RT on a shaker.

All primary and secondary antibodies were diluted in 5% non-fat dry milk in PBS-T. Two primary rabbit anti-RFX2 antibodies were successfully tested and used at a 1:1000 dilution (Atlas Antibodies HPA048969 – Fig. 3B, Additional file 3: Fig. S1A; Thermo Fisher Scientific PA5-61850 – data not shown). Mouse anti-active-β-catenin was used at 1:2000 (Sigma-Aldrich 05–665; Fig. 8B-D, Additional file 3: Fig. S1B). Rabbit anti-GAPDH was used as housekeeping reference protein antibody (Invitrogen PA1-987; 1:1000). The secondary antibodies were horseradish peroxidase (HPR)-conjugated (Sigma-Aldrich A0545, anti-rabbit 1:10,000; GE Healthcare NA931V, anti-mouse 1:5000). Protein expression was detected after 3 min incubation with a chemoluminescent substrate (GE Healthcare RPN2106 for GAPDH; Thermo Fisher Scientific 80196 for active-β-catenin; Thermo Fisher Scientific 34095 for RFX2). Images were taken with the ChemiDoc Touch Imaging System (Bio-Rad) and bands were quantified with the Fiji software [92].

### Isolation of total RNA

Total RNA was extracted from wild type and RFX2 -/- LUHMES cells and column-purified using an RNeasy mini kit (Qiagen 74104) following the manufacturer’s protocol. RNA integrity was checked in 1% agarose gel electrophoresis (Sigma-Aldrich A9539) and concentration and purity were assessed with a Nanodrop ND-100 (Thermo Fisher Scientific). For library preparation and transcriptome analysis, an additional DNase step (Qiagen 79254) was introduced during RNA isolation. RNA integrity values were evaluated using an Agilent Tech 2200 Tape Station (RIN value > 8; KI Bioinformatics and Expression Analysis core facility; https://ki.se/en/bionut/bea-core-facility). RNA concentration was determined with a Qubit 3.0 Fluorometer (Thermo Fisher Scientific). The samples were then diluted to 10 ng/μl (± 0.5 ng/µl) in nuclease-free water before proceeding with the RNA-seq library preparation.

### cDNA synthesis and quantitative real-time PCR (qRT-PCR)

1 μg of total RNA was used to synthesize cDNA using a 1:1 mix of oligo (dT) and random hexamer primers of a RevertAid H Minus First Strand cDNA Synthesis Kit (Thermo Fisher Scientific K1631). The cDNA product was further diluted 1:4 in nuclease-free water and 2 μl were used in a 15 μl total reaction for qRT-PCR (7500 Fast Real-Time PCR instrument; Applied Biosystems), using SYBR green as nucleic acid stain (Roche 4913850001). To specifically quantify mRNA expression, both forward and reverse primers (Additional file 1: Table S6) were designed on consecutive exons and, during the qRT-PCR run, a melting curve analysis was performed to ensure specific amplification of the respective gene of interest. Relative mRNA levels were evaluated using the 2^−ΔΔ Ct^ method and GAPDH was used as housekeeping gene to normalize the values [96].

### siRNA-mediated gene knockdown

To knock down the expression of the essential ciliary genes *IFT88* and *IFT172*, we used a siRNA approach to interfere with mRNA translation. As a non-specific control, fluorescently labeled and scrambled non-silencing siRNA was used (Qiagen 1027292), while to target the genes of interest, pooled mixtures of four different gene-specific siRNAs (Qiagen 1027292), respectively, were used to maximize experimental efficiency. 3.75 × 10^5^ LUHMES cells were seeded in single wells (6-well plate size), respectively, and grown overnight. On the next day (d0), differentiation medium was added, and cells were transfected with lipofectamine RNAi MAX reagent (Invitrogen 13778030), following the manufacturer´s instructions. All siRNAs were used at a final concentration of 150 nM diluted at a 1:1 ratio with lipofectamine and cells were incubated with siRNA-lipid complexes for 72 h (until d3) prior to RNA isolation.

### Drug treatment assays (Wnt-C59, Wnt-3a; SAG, cyclopamine)

3.75 × 10^5^ LUHMES cells were seeded in a pre-coated 6-well plate (Corning 353046) for growth overnight before switching to the differentiation culture medium (d0). Wnt-C59 (Tocris 5148), an inhibitor of WNT signaling [53], was used at 2 µM for a 72 h incubation step (from d0 until d3 of neuron differentiation). The WNT signaling pathway activator Wnt-3a [97] (R&D Systems 5036-WN-010) was instead supplied at 200 ng/ml for 24 h (on d1), before adding another 200 ng/ml on d2, followed by 24 h incubation (until d3, 48 h total incubation). As control, LUHMES cells were cultured following the same differentiation and drug incubation procedures, whereby only 0.2% DMSO and PBS (0.1% BSA) were added, as these were used as vehicle for Wnt-C59 and Wnt-3a, respectively. The modulation of the SHH signaling pathway using the agonist SAG (Merck 566660) or the antagonist cyclopamine (Sigma-Aldrich C4116) was carried out following the experimental procedure previously described [26].

### STRT RNA-seq library preparation and sequencing

We used 20 ng of RNA to generate two 48-plex RNA-seq libraries employing a modified STRT method with unique molecular identifiers (UMIs) [46]. Briefly, RNA samples were placed in a 48-well plate, and a universal primer, template-switching oligonucleotides and a well-specific 6-bp barcode sequence (for sample identification) were added to each well of the plate [98]. The synthesized cDNAs from these samples were then pooled into one library and amplified by single-primer PCR using a universal primer sequence. STRT library sequencing was carried out with an Illumina NextSeq 500 System, High Output (75 cycles), at the Biomedicum Functional Genomics Unit (FuGU), University of Helsinki, Finland (https://www.helsinki.fi/en/infrastructures/genome-analysis/infrastructures/biomedicum-functional-genomics-unit).

### STRT RNA-seq data preprocessing

The raw STRT sequencing data in the Illumina Base Call Format were demultiplexed and processed using the STRT2 pipeline [45] (https://github.com/my0916/STRT2). Reads were aligned to the human reference genome hg19, the human ribosomal DNA unit (GenBank: U13369) and ERCC spike-ins (SRM 2374) using GENCODE (v28) transcript annotation by HISAT2 (v2.2.1) [99] with the option ‘–dta’. For gene-based analyses, uniquely mapped reads within the 5’ UTR or 500 bp upstream of protein-coding genes were counted using Subread feature Counts (v2.0.0). For transcript far 5′ end (TFE) based analyses, the mapped reads were assembled using StringTie (v2.1.4) [100], and mapped reads within the first exons of the assembled transcripts were counted as previously described [101]. FASTQ files after exclusion of duplicated reads were deposited in the ArrayExpress database at EMBL-EBI (https://www.ebi.ac.uk/biostudies/arrayexpress/studies/E-MTAB-11546): accession number E-MTAB-11546 [102]. Numbers of total and mapped reads for each sample are summarized in Additional file 1: Table S2.

### Downstream expression analysis

Among the 80 samples from wild type and RFX2 -/- LUHMES cells and neurons, two samples were excluded as outliers due to extremely low numbers of total read counts. After filtering out lowly expressed genes and ERCC spike-ins, differential expression analysis was performed with the R (v4.0.3) package DESeq2 (v1.30.1) [103]. Genes or TFEs with an adjusted P-value of less than 0.05 were considered as significantly differentially expressed. Gene ontology (GO) term enrichment analysis was performed using the R package enrichR (v3.0) [104]. Motif enrichment on differentially expressed TFEs was analyzed using the command findMotifsGenome.pl from HOMER (v4.11) [105] with the option ‘-size -300,100 -mask’, using all detected TFEs as background. Principal component analysis (PCA) was carried out using the 500 genes with the highest variance across all 78 samples with the DESeq2 PCAplot function. Time-course clustering was performed using the R package maSigPro (v1.62.0) [106]. Gene set enrichment analysis (GSEA) was performed with the R package fgsea [107], where genes pre-ranked based on their *P*-values and fold changes were compared with the gene set of 686 ciliary genes from the SYSCILIA gold standard (SCGSv2) [43]. Integrative analysis with the fetal midbrain single-cell RNA-seq data (GSE76381) [48, 108] was carried out with the R package SingleR (v1.4.1) [109], using the log2-normalized expression levels of the LUHMES STRT RNA-seq data as the reference data set.

The abbreviations for (neuronal) cell types (comparative analyses presented in Fig. 6B) are the following: OMTN, oculomotor and trochlear nucleus; Sert, serotonergic neurons; Gaba, GABAergic neurons; NbGaba, GABAergic neuroblasts; DA, dopaminergic neurons; NbML, mediolateral neuroblasts; RN, red nucleus; NbM, medial neuroblast; NProg, neuronal progenitor; Prog, progenitor medial floor plate (FPM), lateral floor plate (FPL), basal plate (BP), midline (M); Rgl, radial glia-like cells; OPC, oligodendrocyte precursor cells; Mgl, microglia; Peric, pericytes; Endo, endothelial cells. The order of these (neuronal) cell types follows the same order as originally presented in La Manno et al. [48].

### Supplementary Information

**Additional file 1.**

**Additional file 2.**

**Additional file 3.**

## Data Availability

We have submitted our STRT sequencing (transcriptome) data to the EMBL – European Bioinformatics Institute (EBI) BioStudies ArrayExpress database (https://www.ebi.ac.uk/biostudies/arrayexpress/studies/E-MTAB-11546) where these data are publicly available for review [102].

## Abbreviations

A.U.: Arbitrary unit
AP-1: Activator protein 1
bFGF: Basic fibroblast growth factor
DBD: DNA binding domain
DVL: Dishevelled
GO: Gene ontology
gRNA: Guide RNA
GSEA: Gene set enrichment analysis
IFT: Intraflagellar transport
KD: Knockdown
LUHMES: Lund human mesencephalic neuronal cell line
MTOC: Microtubule-organizing center
NPHP: Nephronophthisis
PAM: Proto-spacer adjacent motif
PCA: Principal component analysis
PCP: Planar cell polarity
PyT: Pythagorean theorem
qRT-PCR: Quantitative real-time PCR
RT: Room temperature
SAG: Smoothened agonist
SCGSv2: SYSCILIA consortium gold standard ciliary gene list (version 2)
STRT RNA-seq: Single-cell tagged reverse transcription RNA-sequencing
TCF/LEF: T-cell factor/lymphoid enhancer factor
TF: Transcription factor
TFE: Transcript far end
UMI: Unique molecular identifier
WT: Wild type
