# Supplementary material for: Primary cilia promote the differentiation of human neurons through the WNT signaling pathway
Source: BMC Biol. 2024 Feb 27;22:48. doi: 10.1186/s12915-024-01845-w (PMC10900739; doi:10.1186/s12915-024-01845-w)
Supplement: Supplementary file 2 — Additional file 2. [file 12915_2024_1845_MOESM2_ESM.zip › Additional.file.2.Fig.Legends.pdf]

Fig. S1 (related to Fig. 3): Mutation of ciliogenic *RFX2* does not affect cell proliferation, or neuron-type population characteristics.

(A) Schematic illustration of the transcription factor (TF) *RFX2* gene structure, including start codon (ATG), exons and introns, and stop codon (TGA). Guide RNAs (gRNA) for CRISPR/Cas9 mutagenesis were designed to bind to exon 5 sequences, upstream of the DNA binding domain (DBD), encoded by exons 6 and 7. PAM = proto-spacer adjacent motif. **(B)** qRT-PCR quantification demonstrates strong downregulation of *RFX2* gene expression in LUHMES RFX2-/- as compared to WT, throughout the entire neuron differentiation process (d0-d6). **(C)** CRISPR/Cas9 mutagenesis causes DNA sequence deletions: allele a (22 bp) and allele b (43 bp). **(D)** These CRISPR/Cas9 deletions result in translational amino acid frame shifts and premature stop codons, destroying the RFX2 DBD, essential for TF function. **(E)** There are no growth rate differences between LUHMES WT and RFX2 -/- backgrounds, neither in growth or proliferation medium (0h-72h) (Top), nor in growth or proliferation (0h-24h) and neuron differentiation media (24h-96h) (Bottom). **(F)** Schematic representation of differentiating neuron types in LUHMES cell culture populations: neurons with a single main neurite (emerging axon – unipolar); neurons with two main neurites (emerging axon and future dendrite – bipolar); and neurons with a single main neurite and multiple secondary neurites (emerging axon and future dendrites – multipolar). Quantification of neuron types in LUHMES WT (n=298-383) and RFX2 -/- backgrounds (n=74-337) reveals a vast majority of bipolar neurons in the cell culture populations and no relevant anatomical differences. **(G)** Neurons at differentiation stage 2a display a significantly larger diameter of the emerging axon than neurons at stages 2b and 3 (n=19-33). **(H)** Axon elongation between differentiation stages 2a, 2b and 3 (n=28-50).

Mean values are shown ± s.e.m **(B, G)**, ± s.d. **(E-F)** and displayed as Box and Whisker plots (min to max) **(H)**. The results are from a minimum three independent experiments with two technical replicates each. We conducted regular one-way **(G-H)** and two-way ANOVA analyses (not repeated measures) **(B, E-F)** with multiple comparisons (Bonferroni's test) between groups. $^{**}p<0.005$; $^{***}p<0.0005$; $^{****}p<0.0001$.

Fig. S2 (related to Fig. 4 and 7): Joint statistical analysis of axon branching of LUHMES WT and RFX2 -/- stage 3 neurons.

(A) Axons of ciliated WT neurons are more branched than their non-ciliated counterparts and as branched as the axons of ciliated and non-ciliated RFX2 -/- neurons at all time points (d1-d3). Axons of non-ciliated RFX2 -/- neurons are significantly more branched than axons in non-ciliated WT neurons, especially at d1 and d3. This aspect is confirmed in control neurons (vehicle treatment) in the corresponding experiments using the WNT signaling pathway inhibitor Wnt-C59 **(B)**.

Mean values are shown ± s.d. The results are from a minimum of three independent experiments with a minimum of two technical replicates each. We conducted regular two-way ANOVA analyses (not repeated

measures) with multiple comparisons (Bonferroni's test) between groups. **$p<0.005$; ***$p<0.0005$; ****$p<0.0001$.

Fig. S3 (related to Fig. 6): Clustering of variable genes between WT and RFX2 -/- samples throughout LUHMES neuron differentiation (d0-d6).

(Left) 3,476 genes with a significantly variable expression between LUHMES WT and RFX2 -/- backgrounds were clustered into three clusters based on their expression patterns during the neuron differentiation process (d0-d6). Lines are local polynomial regression fittings of the scaled expression of the genes in each cluster, depicted in blue (WT) and in red (RFX2 -/-). Cluster 3 is also shown in Figure 6C. **(Right)** Gene ontology (GO) enrichment analysis of the genes in each cluster. Red dashed lines represent adjusted p-value = 0.05.

Fig. S4 (related to Fig. 6): Gene expression profiles of LUHMES WT and RFX2 -/- STRT RNA-seq samples during neuron differentiation (d0-d6).

Relevant **(A)** neuronal marker genes, **(B)** neuronal function-related genes, **(C)** ciliary genes, **(D)** WNT/β-catenin signaling pathway genes and the *RFX2* gene are depicted in blue (WT) and in red (RFX2 -/-).

Fig. S5 (related to Fig. 6): GO and TF binding motif enrichment analyses of downregulated genes in LUHMES RFX2 -/- STRT RNA-seq-samples (d0-d6).

(A) Gene ontology (GO) terms significantly enriched on at least two time points are shown. Colors represent -log10 (adjusted p-value) and circle sizes represent the number of genes in each category. The total number of significantly downregulated genes on each day of neuron differentiation is shown in parenthesis. **(B)** TF binding motif enrichment analyses for neuron differentiation days 0-6 are shown. Days 2 and 3 are also shown in Figure 6D. Only the top five significantly enriched motifs are listed.

Fig. S6 (related to Fig. 7 to 9): Modulation of the canonical SHH signaling pathway in LUHMES WT and RFX2 -/- neurons.

(A) Expression of canonical SHH target genes (*GLI1, HHIP, PTCH1, PTCH2*) in LUHMES WT and RFX2 -/- neurons (d3) is either upregulated similarly upon treatment with Smoothened agonist (SAG) or downregulated similarly using cyclopamine as inhibitor, as compared to control neurons (vehicle). **(B)** Time course STRT RNA-seq data show a downregulation of *GLI1* expression, very low *HHIP* expression and steady expression of the canonical SHH pathway inhibitors *PTCH1* and *PTCH2* during neuron differentiation (d1-d6).

Mean values are shown ± s.e.m. The results are from a minimum of three independent experiments with two technical replicates each. We conducted regular two-way ANOVA analyses (not repeated measures)

with multiple comparisons (Bonferroni's test) between groups. *$p<0.05$; **$p<0.005$; ***$p<0.0005$; ****$p<0.0001$.

Fig. S7 (related to Fig. 8): Functional primary cilia mediate the reduction of canonical WNT signaling pathway activation.

(A) General activation of canonical WNT signaling: In the presence of a WNT ligand (in green), Dishevelled (DVL) protein inhibits the β-catenin-targeting destruction complex (GSK-3β, AXIN, CK1α, APC), promoting a cytoplasmic accumulation of active β-catenin (non-phosphorylated) and its nuclear translocation to activate TCF/LEF transcription factors, in turn affecting target gene expression (comparable to Figure 8C-D; non-ciliated). In the absence of a WNT ligand (in red), the destruction complex is activated and leads to β-catenin inactivation (phosphorylated) and degradation. **(B)** A functional primary cilium finetunes the balance between canonical and non-canonical WNT signaling, both of which can simultaneously be active. A ciliary protein complex (Nephronophthisis; NPHPs) regulates partial DVL cytoplasmic degradation, reducing its inhibition of the destruction complex. As a result, the cytoplasmic accumulation of active β-catenin and subsequent canonical WNT activation is reduced, indirectly promoting the activation of the non-canonical planar cell polarity (PCP) WNT pathway involved in cytoskeletal remodeling (comparable to Figure 8C; ciliated). **(C)** When cilium function is altered, DVL largely inhibits the destruction complex leading to a canonical WNT overactivation that outbalances the non-canonical route (comparable to Figure 8D; ciliated).
