# Supplementary figures and images for: Primary cilia promote the differentiation of human neurons through the WNT signaling pathway
Source: BMC Biol. 2024 Feb 27;22:48. doi: 10.1186/s12915-024-01845-w (PMC10900739; doi:10.1186/s12915-024-01845-w)

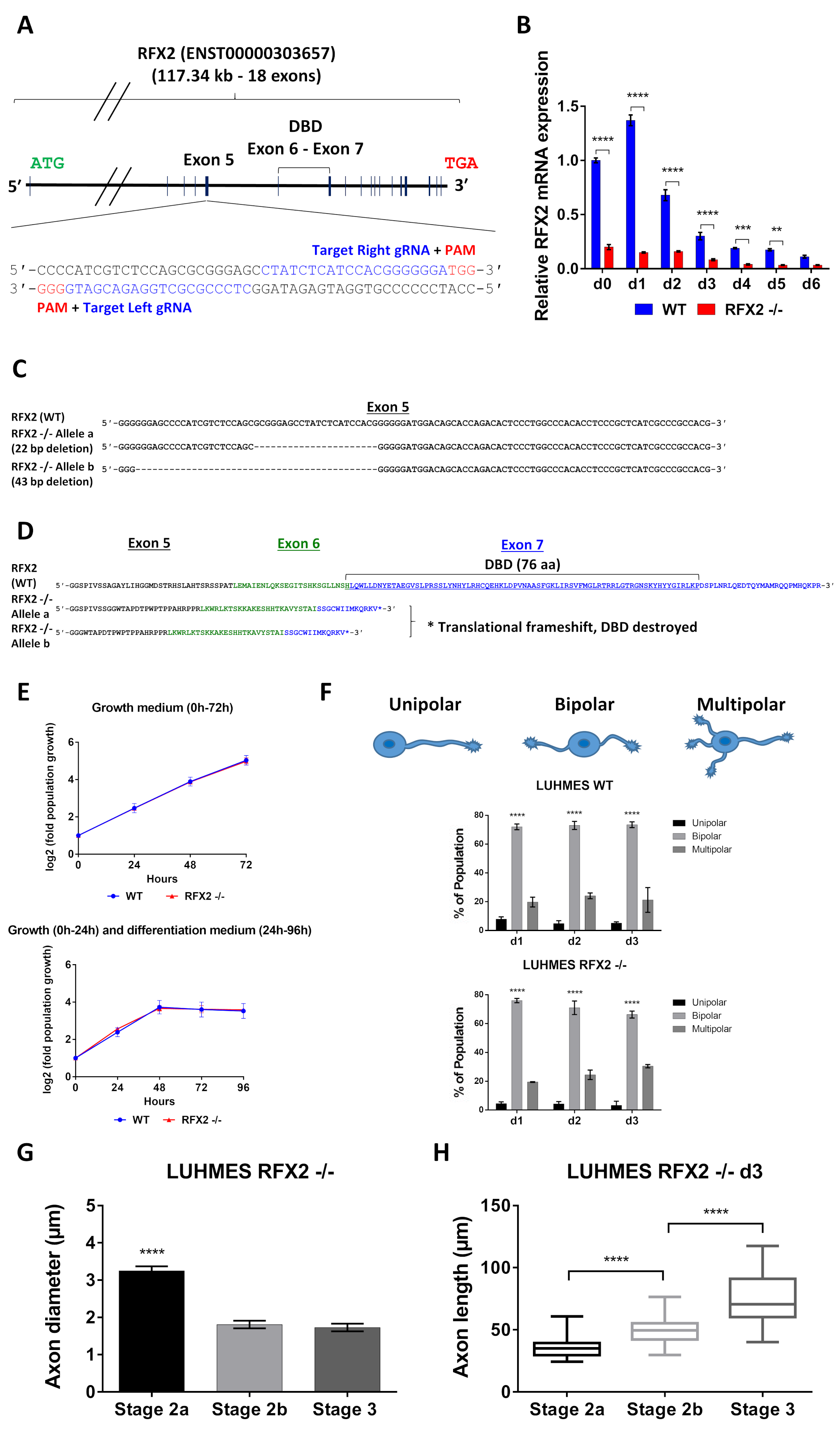

Supplement: Supplementary file 2 — Additional file 2. [file 12915_2024_1845_MOESM2_ESM.zip › Additional.file.2.Fig.S1.AC.et.al.png]

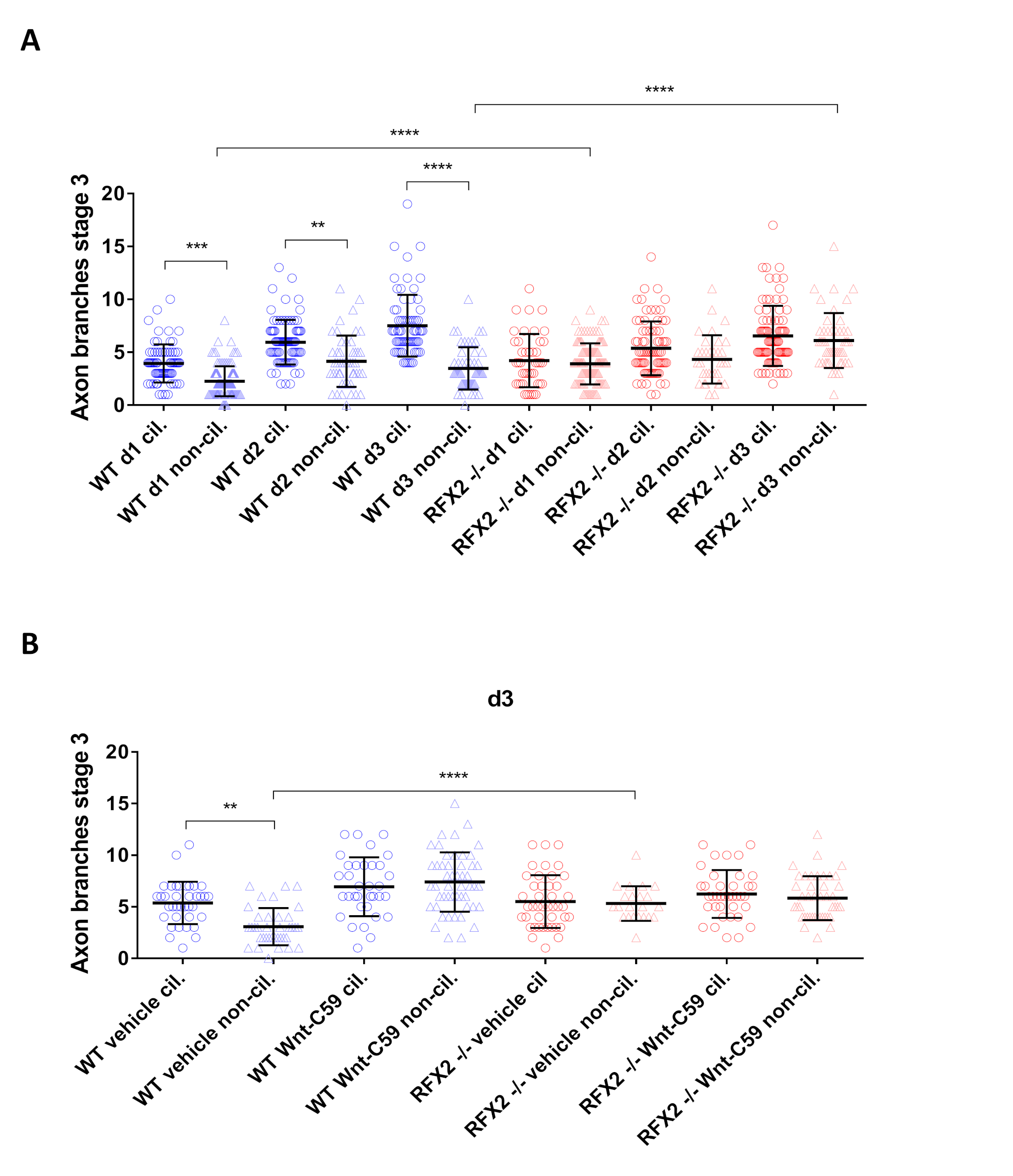

Supplement: Supplementary file 2 — Additional file 2. [file 12915_2024_1845_MOESM2_ESM.zip › Additional.file.2.Fig.S2.AC.et.al.png]

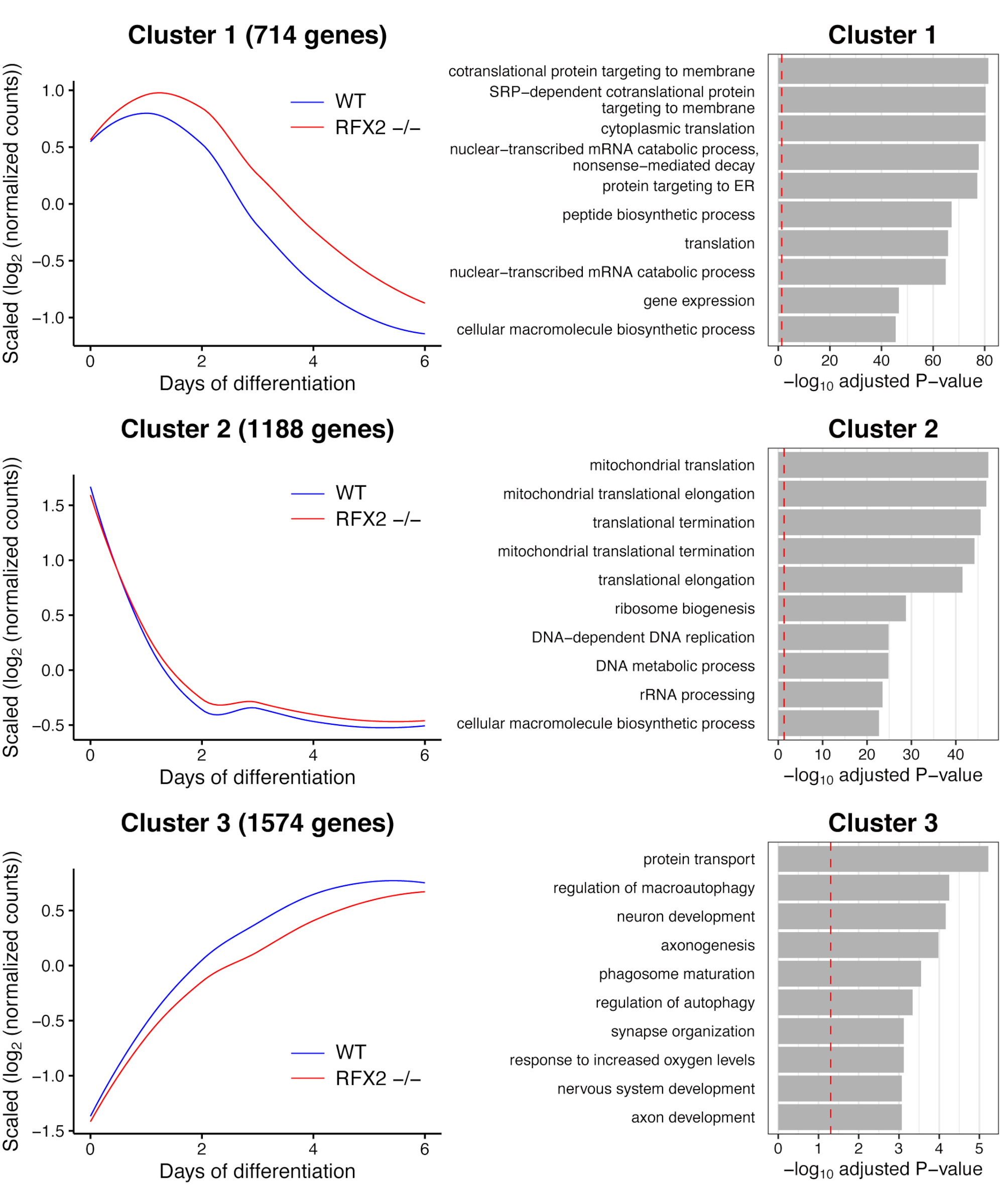

Supplement: Supplementary file 2 — Additional file 2. [file 12915_2024_1845_MOESM2_ESM.zip › Additional.file.2.Fig.S3.AC.et.al.png]

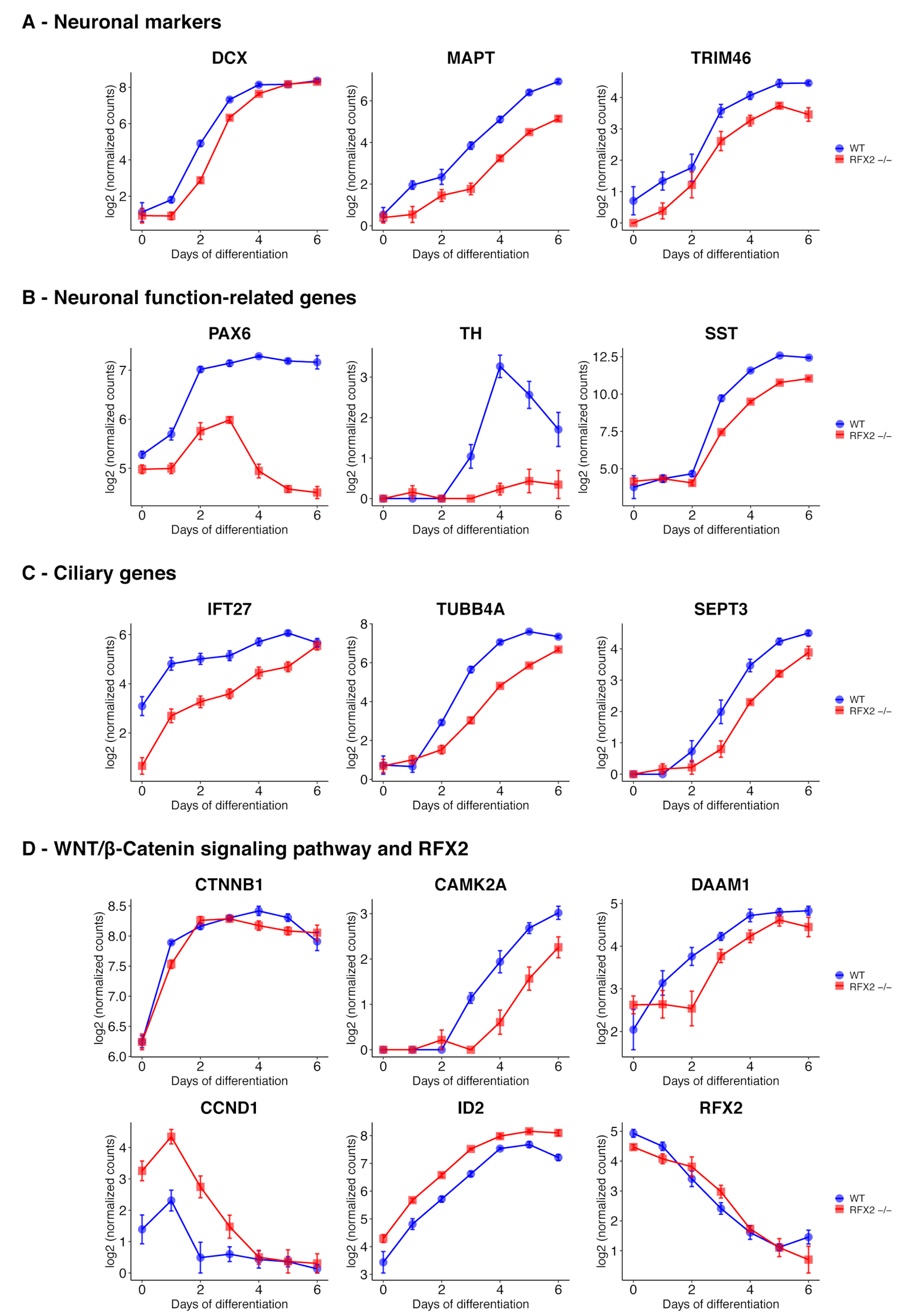

Supplement: Supplementary file 2 — Additional file 2. [file 12915_2024_1845_MOESM2_ESM.zip › Additional.file.2.Fig.S4.AC.et.al.png]

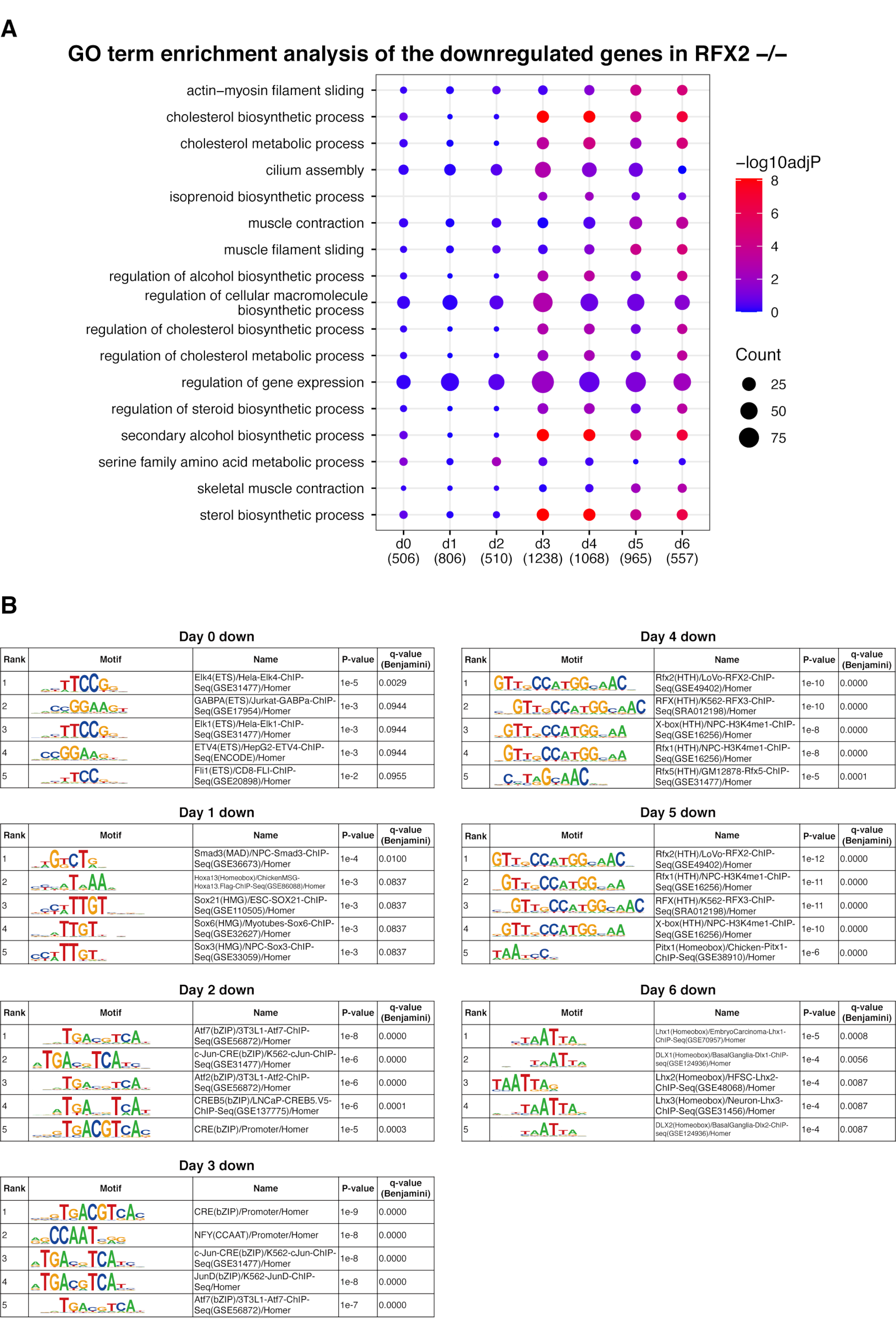

Supplement: Supplementary file 2 — Additional file 2. [file 12915_2024_1845_MOESM2_ESM.zip › Additional.file.2.Fig.S5.AC.et.al.png]

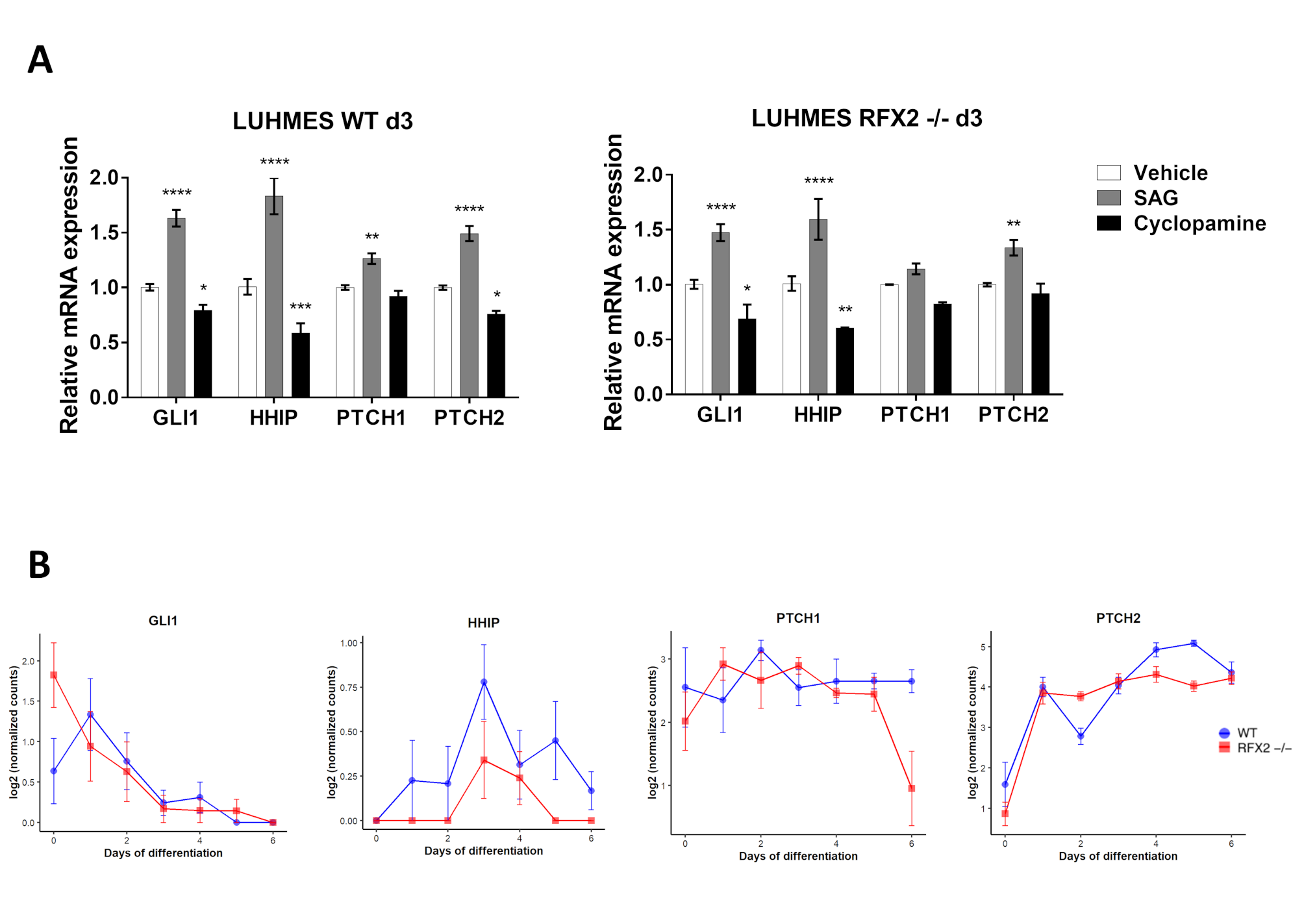

Supplement: Supplementary file 2 — Additional file 2. [file 12915_2024_1845_MOESM2_ESM.zip › Additional.file.2.Fig.S6.AC.et.al.png]

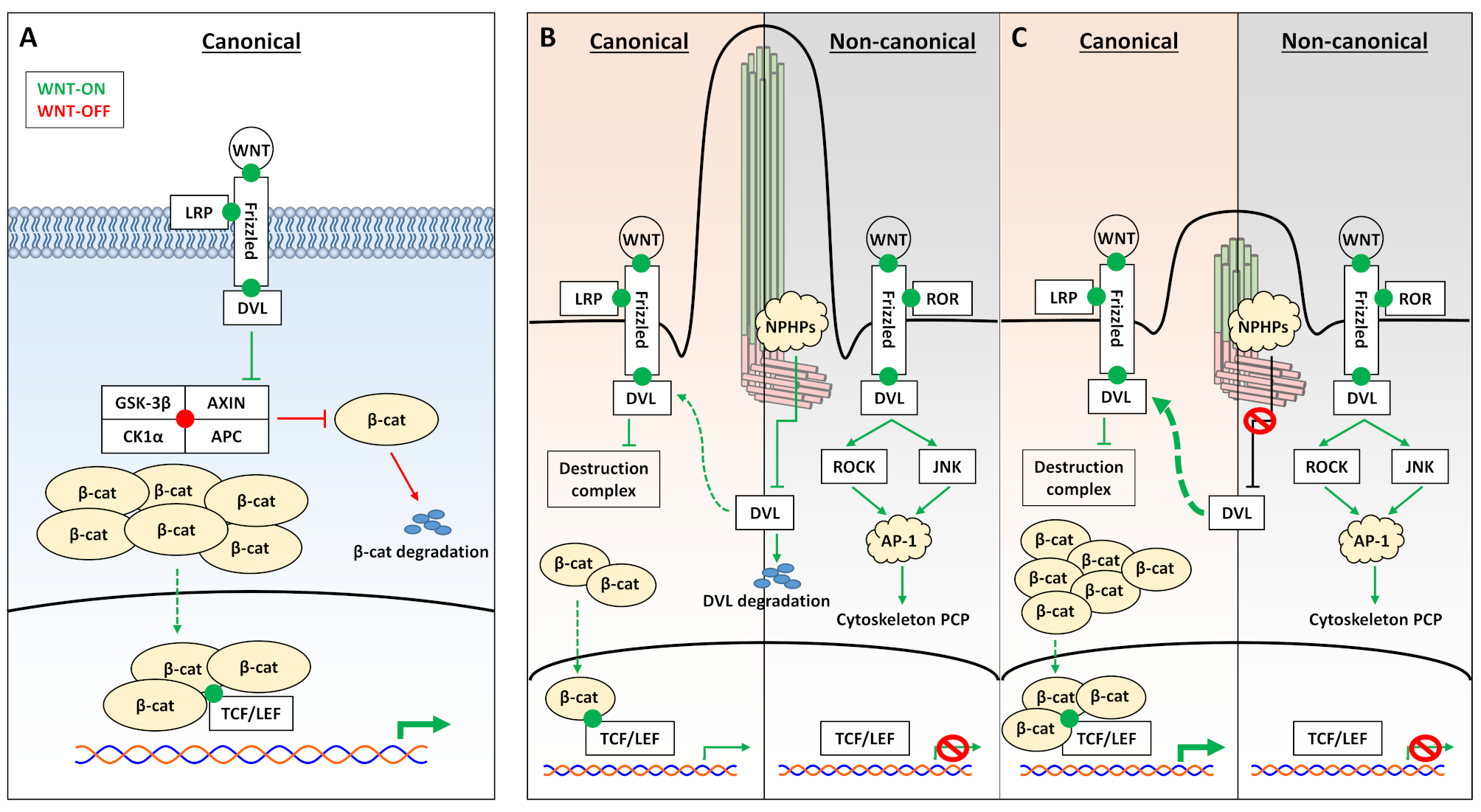

Supplement: Supplementary file 2 — Additional file 2. [file 12915_2024_1845_MOESM2_ESM.zip › Additional.file.2.Fig.S7.AC.et.al.png]

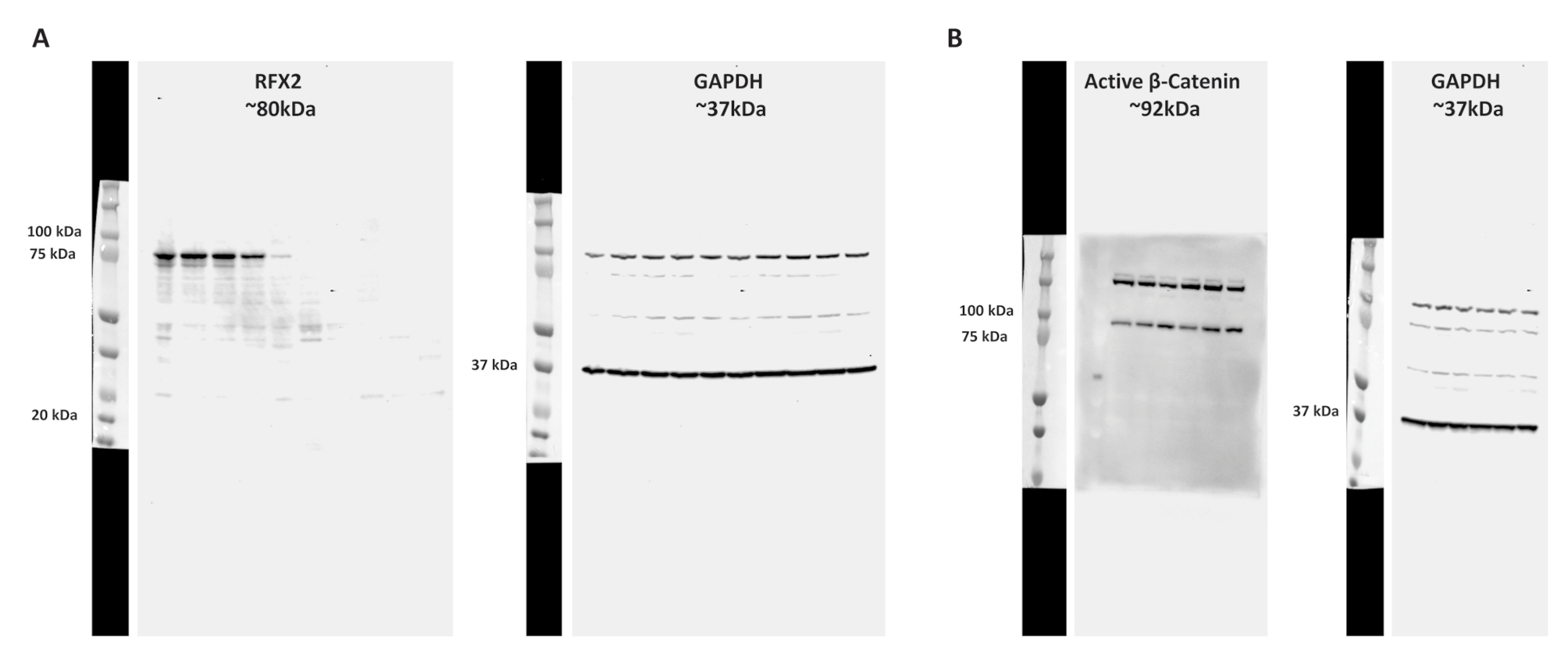

Supplement: Supplementary file 3 — Additional file 3. [file 12915_2024_1845_MOESM3_ESM.png]
